# PLA2G16‐Mediated Tetracosatetraenoic Acid Rewires Fatty Acid Oxidation to Impair CD8^+^ T Cell Immune Function in Promoting Breast Cancer Lung Metastasis

**DOI:** 10.1002/advs.202510224

**Published:** 2025-11-16

**Authors:** Yubi Gan, Die Meng, Lei Lang, Jing Luo, Peijin Dai, Chao Chang, Zexiu Lu, Yuetong Guo, Rui Wang, Shanchun Chen, Xi Tang, Yixuan Hou, Dongmei Tan, Manran Liu

**Affiliations:** ^1^ Key Laboratory of Clinical Laboratory Medical Diagnostics Chinese Ministry of Education Chongqing Medical University Chongqing 400016 China; ^2^ Western Institute of Digital Intelligent Medicine Chongqing 401329 China; ^3^ Department of Pathology College of Basic Medicine Molecular Medicine Diagnostic and Testing Center Chongqing Medical University Department of Pathology The First Affiliated Hospital of Chongqing Medical University Chongqing 400016 China; ^4^ Department of Laboratory Medicine The First Affiliated Hospital of Chongqing Medical University Chongqing 400016 China; ^5^ Experimental Teaching Center of Basic Medicine Science Chongqing Medical University Chongqing 400016 China; ^6^ Laboratory Animal Center Chongqing Medical University Chongqing 400016 China

**Keywords:** lipid metabolism, lung metastasis, PLA2G16, T cell, tetracosatetraenoic acid

## Abstract

Tumor‐related metabolites in the tumor microenvironment may induce immune dysfunction, leading to malignant progression and metastasis of tumors. Here, it is demonstrated that tumoral PLA2G16, a phospholipase catalyzes phospholipids to generate free fatty acid (FFA) or lysophosphatidic acid (LPA), is an important contributor to triple‐negative breast cancer (TNBC) lung metastasis in an immune‐dependent pattern by improving tetracosatetraenoic acid (C24:4 (n‐6)) accumulation in the early metastatic niche of lung and impairing immune function of pulmonary CD8^+^ T cells. C24:4 (n‐6) induces nuclear import of PPARα in pneumal CD8^+^ T cells, which regulates the transcription of *Cpt1a, Dgat1, Cd36*, and *Fabp1*, leading to the activation of fatty acid oxidation (FAO). The robust FAO results in suppression of CD8^+^ T cells. Genetically depleting PPARα in mice, pharmacologically inhibiting C24:4 (n‐6)‐induced PPARα in the nucleus or directly suppressing PPARα activity effectively attenuates PLA2G16‐C24:4 (n‐6) axis‐based immune dysfunction of CD8^+^ T cells and their according anti‐tumor activities. These results imply that PLA2G16‐mediated C24:4 (n‐6) accumulation in the lung acts as a metabolic disorder to CD8^+^ T cell antitumor activity and highlights a critical role of PLA2G16 in promoting TNBC lung metastasis. Targeting PLA2G16 and combination with anti‐PD‐1‐based immunotherapy may be an effective strategy for clinical tumor immunotherapy.

## Graphical abstract

Illustrating how tumor‐derived PLA2G16 regulates tetracosatetraenoic acid (C24:4 (n‐6)) accumulation and CD8^+^ T cell activation via the C24:4‐PPARα‐FAO axis in early metastatic niches, thereby promoting lung metastasis of triple‐negative breast cancer.

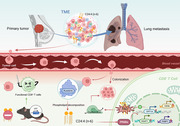

## 1 Introduction

Immune checkpoint blockade (ICB), including programmed cell death protein 1 (PD‐1)/programmed death ligand 1 (PD‐L1) inhibitors, has revolutionized cancer care and is quickly taking the lead for cancer treatment.^[^ ^1^ ^]^ Despite the remarkable efficacy of ICB therapy in multiple cancer types, only a small percentage of patients with breast cancer (BC), especially Triple‐negative breast cancer (TNBC), which has comparatively modest baseline T cell infiltrates, have benefited from ICB treatment.^[^ ^2^, ^3^ ^]^ PD‐1 blockade exhibits an overall response rate of 18.5%, even in patients with breast cancer that is positive for PD‐L1.^[^ ^4^ ^]^ Increasing CD8^+^ T cell infiltration into tumors can improve cancer immunotherapy in BC.^[^ ^5^, ^6^ ^]^ Identifying the determinative crosstalk between tumor and immune microenvironment is essential to convert the “cold” breast tumors into “hot” immunogenic tumors and improve the efficacy of clinical immunotherapy.

Breast cancer is the most common malignant tumor in women and the second‐leading cause of cancer‐related deaths worldwide.^[^ ^7^ ^]^ Previous studies have acquired a great progress in malignant behavior, progression, and therapeutic strategies of primary breast cancer.^[^ ^8^, ^9^ ^]^ However, A growing number of studies provide evidence that the capacity of cancer cells disseminating to secondary sites and forming metastases is the main cause of death in BC.^[^ ^4^, ^10^ ^]^ Significant discoveries in the field of tumor metastasis indicate the interaction between tumor cells and the immune microenvironment in metastatic organs, which is essential for tumor cells to evade immune surveillance and endure in an unfamiliar and hostile environment.^[^ ^11^ ^]^ Owing to heterogeneity and high active energy metabolism in tumor cells, the tumor microenvironment (TME) typically exhibits hypoxia, acidity, nutrient depletion, and accumulation of tumor‐related metabolites, with lipid enrichment being a frequent observation.^[^ ^12^, ^13^ ^]^ The function of tumor‐infiltrating immune cells can be influenced by complex metabolite components in the TME.^[^ ^14^, ^15^, ^16^ ^]^ Moreover, the metabolic flexibility of immune cells and their adaptation to TME are key to their functional specialization.^[^ ^17^, ^18^ ^]^ Several studies have reported the impact of various lipids, especially the free fatty acids (FFAs), on T cell proliferation, activation, and differentiation in terms of metabolic intermediates, membrane components, or signaling molecules.^[^ ^19^, ^20^, ^21^, ^22^ ^]^ However, it remains unclear whether and how specific lipid metabolites accumulation regulated by tumor cells may contribute to the functional modification of CD8^+^ T cells infiltrating the metastatic organs.

PLA2G16 belongs to phospholipase A2 family group XVI, also referred to as AdPLA2 (adipose‐specific PLA2), H‐REV‐107, HRASLS3 (Ha‐RAS‐like suppressor 3) and PLAAT3. Before the phospholipase activity of PLA2G16 was discovered, it was considered as a tumor suppressor.^[^ ^23^, ^24^, ^25^ ^]^ Interestingly, as a phospholipase, PLA2G16 catalyzes hydrolysis from phospholipids sn‐2 ester bond and generates FFAs or lysophosphatidic acid (LPA),^[^ ^26^ ^]^ whereas FFAs are critical signal transduction molecules and metabolism regulators in tumor initiation and progression.^[^ ^27^, ^28^ ^]^ Furthermore, it is possible that PLA2G16 contributes to tumor growth through altering the metabolic pathways, as PLA2G16 null animals are resistant to obesity caused by a high‐fat diet or leptin deficit.^[^ ^24^ ^]^ Nevertheless, whether PLA2G16‐mediated lipid metabolism contributes to tumor progression through interactions with the microenvironment has not been reported in tumors.

Here, we found that tumorous PLA2G16 regulates tetracosatetraenoic acid (C24:4 (n‐6)) accumulation and pneumal CD8^+^ T cells activation in early metastatic niches, hence promoting lung metastasis of breast cancer. Overall, our findings emphasize the significance of PLA2G16 in dictating BC lung metastasis by educating CD8^+^ T cells and lay the foundation for enhancing the efficacy of tumor immunotherapy.

## 2 Results

### 2.1 The Enhanced PLA2G16 is Correlated With Clinical Malignant Characteristics in Breast Cancer Patients

**Figure 1:**
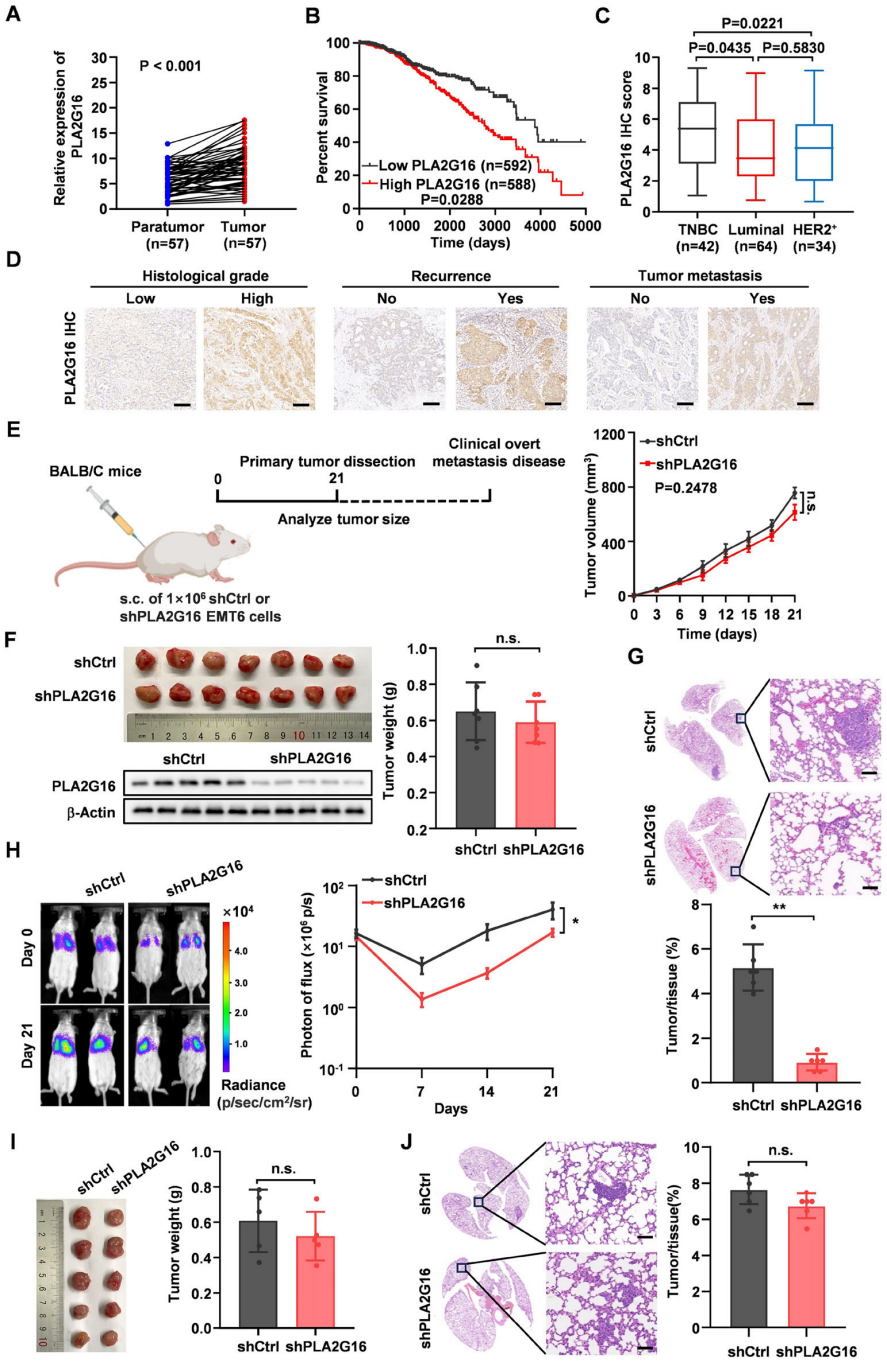
PLA2G16 is upregulated in human breast cancer tissues and associated with breast cancer lung metastasis. A) qRT‐PCR analysis of PLA2G16 expression in a cohort of 57 paired breast cancer tissues and their matched normal tissues (*n* = 57). B) Kaplan–Meier survival plot of breast cancer patients with high or low PLA2G16 expression based on TCGA‐BRCA database. C) IHC score of PLA2G16 stratified by breast cancer subtypes in a cohort of 140 patients (TNBC, *n* = 42; Luminal, *n* = 64; HER2^+^, *n* = 34). D) Representative IHC images of PLA2G16 level in breast cancer tissues. Scale bars, 100 µm. E,F) Experiment scheme (left panel) and tumor burden assessed over time (right panel) are shown (E). Tumors were photographed (upper panel) and weighed (*n* = 7, right panel), and PLA2G16 levels in tumors were assessed by western blot (down panel) (F). G) Representative H&E‐stained sections of lung metastasis (upper panel) and corresponding quantification of metastasis area in mice injected EMT6 with or without PLA2G16 (*n* = 6, down panel). Scale bars: 100 µm. H) Intravenous injection of EMT6 with or without PLA2G16 for lung colonization analysis. Representative images of BLI in each group (left panel) and BLI quantification of the experimental groups (*n* = 4, right panel) are shown. I) BALB/c‐Nude mice were injected with 1×10^6^ MDA‐MB‐231 cells with or without PLA2G16, and 35 days later (unless otherwise indicated), tumors were photographed and weighed (*n* = 5). J) Representative H&E‐stained sections of lung metastasis and corresponding quantification of metastasis area in mice from experiments in (J) (*n* = 5). Scale bars: 100 µm. Data are the mean ± SD. ^*^ *p* < 0.05, ^**^ *p* < 0.01, and ^***^ *p* < 0.001 by paired t test in (A); log rank test in (B); two‐sided unpaired Student's t test in (F), (G), and (I)‐(J); two‐way ANOVA in (E) and (H) or one‐way ANOVA in (C). n.s., not significant. Boxplots display values of minimum, first quartile, median, third quartile, and maximum (C).

The aberrant PLA2G16 expression is closely associated with malignancy of various tumors.^[^ ^30^, ^31^, ^32^ ^]^ In our previous study, PLA2G16 was found to be involved in breast cancer stem cell (BCSC) maintenance.^[^ ^33^ ^]^ Indeed, there was an elevated PLA2G16 level in breast cancer (BC) tissues than their paired non‐tumor tissues (**Figure** **1**A) by measuring PLA2G16 expression in a cohort of 57 paired BC tissues and adjacent normal tissues. BC patients with a high level of PLA2G16 had poorer survival (Figure 1B). To further understand the association of PLA2G16 with the clinicopathological characteristics of BC patients, we probed PLA2G16 expression in another cohort of 140 BC patients and found that PLA2G16 levels were noticeably higher in Triple‐negative breast cancer (TNBC) in comparison with luminal or HER2^+^ subtype (Figure 1C). There was no discernible correlation observed between PLA2G16 expression and tumor size, patient age or vascular invasion (Table S2, Supporting Information). However, the enhanced PLA2G16 expression was associated with clinical tumor grade, recurrence, and distal metastasis (Figure 1D; Table S2, Supporting Information). Interestingly, there was a negative correlation between PLA2G16 expression and metastasis‐free survival of BC patients, especially in TNBC patients, indicating its potential role in the metastasis of TNBC (Figure S1A, Supporting Information). Furthermore, checking PLA2G16 expression in the organotropic metastasis BC cells as previously described,^[^ ^34^ ^]^ we found that PLA2G16 had a stronger expression in preferential lung metastasis cells compared with primary tumors, and liver‐ or brain‐metastasis cells (Figure S1B,C, Supporting Information). The PLA2G16 protein was gradually increased with the selected passages of lung‐preferential metastasis cells (Figure S1D, Supporting Information). All of these findings point to a close link between increased PLA2G16 expression and clinical malignant characteristics, and PLA2G16 may work as a potential factor in driving TNBC lung metastasis.

### 2.2 PLA2G16 Promotes Lung Metastasis of Breast Cancer

To explore the functional role of PLA2G16 on lung metastasis of TNBC, PLA2G16 levels in various human and mouse TNBC cells were assessed by qRT‐PCR and Western blotting analysis (Figure S2A–D, Supporting Information); then, the stable knockdown of endogenous PLA2G16 cell models (e.g., MDA‐MB‐231/PLA2G16 shRNA or EMT6/PLA2G16 shRNA) and ectopic PLA2G16 overexpressing cells (e.g., BT549/PLA2G16 or PY8119/PLA2G16) were established (Figure S2E–F, Supporting Information). Curiously, the silence of PLA2G16 did not impair primary tumor growth in immunocompetent BALB/c mice (Figure 1E,F), but significantly reduced lung‐metastatic burden (Figure 1G,H). In contrast, ectopic PLA2G16 overexpression exacerbated lung metastatic burden in immunocompetent C57BL/6J mice (Figure S2G,H, Supporting Information). However, PLA2G16 knockdown had little effect on primary tumor growth or spontaneous lung metastasis of MDA‐MB‐231 tumors in immunodeficient BALB/c nude mice (Figure 1I,J). These data suggest that PLA2G16 potentially acts in its pro‐metastatic functions in an immune‐dependent pattern.

### 2.3 PLA2G16 Primes Survival of Disseminated Tumor Cells During the Early Stages of Metastatic Colonization

Based on the aforementioned findings, PLA2G16 knockdown‐induced reduction of BLI signal in lungs was pretty noticeable within 7 days (Figure 1H), suggesting a potential function of PLA2G16 in the early metastatic stage. The frequency of circulating tumor cells (CTCs) reflects the capacity of tumor cells in invasion, intravasation into blood vessels, and survival in circulation.^[^ ^35^ ^]^ Thus, we checked CTCs in tumor‐burden mice injected with PLA2G16‐knockdown EMT6 cells and control cells. However, there were comparable numbers of CTCs in the tumor‐burden mice injected with PLA2G16‐knockdown and control tumor cells, indicating that loss of PLA2G16 has less effect on CTCs (Figure S3A, Supporting Information). By IF staining and the daily lung BLI signals, we then assessed the seeding and colonization capacity of BC cells in mouse lungs. Here, seeding is strictly referred to the arrival of disseminated tumor cells (DTCs) at the mouse lung tissue; and colonization, in contrast, encompasses the subsequent phase where DTCs obtain survival ability and initiate proliferation to ultimately form macroscopic metastases in the lung. Within 24 h after intravenous injection, there was almost no difference in the cell numbers of DTCs between the control and PLA2G16‐silenced groups (Figure S3B, Supporting Information, left panels), indicating that PLA2G16 has no effect on DTC seeding in the lung. However, PLA2G16 knockdown resulted in a reduction of colonized DTCs or metastases in lungs at the initial time (48 h to 7 days) (Figure S3B,C, Supporting Information). Consistently, no difference of Ki67^+^ tumor cells between PLA2G16‐silenced and control groups (Figures S3D, Supporting Information), while more of cleaved caspase three positive cells were observed in the PLA2G16‐deficient metastases compared to the control metastases (Figure S3E, Supporting Information), further to indicate that PLA2G16 affects DTC survival in the early stage of lung metastasis. Consistently, ectopic PLA2G16 overexpression significantly increased DTC survival in the early colonization stage (Figure S3F,G, Supporting Information).

### 2.4 Tumoral PLA2G16 Suppresses Function of Lung Infiltrating CD8^+^ T Cells to Facilitate Breast Cancer Metastasis

**Figure 2:**
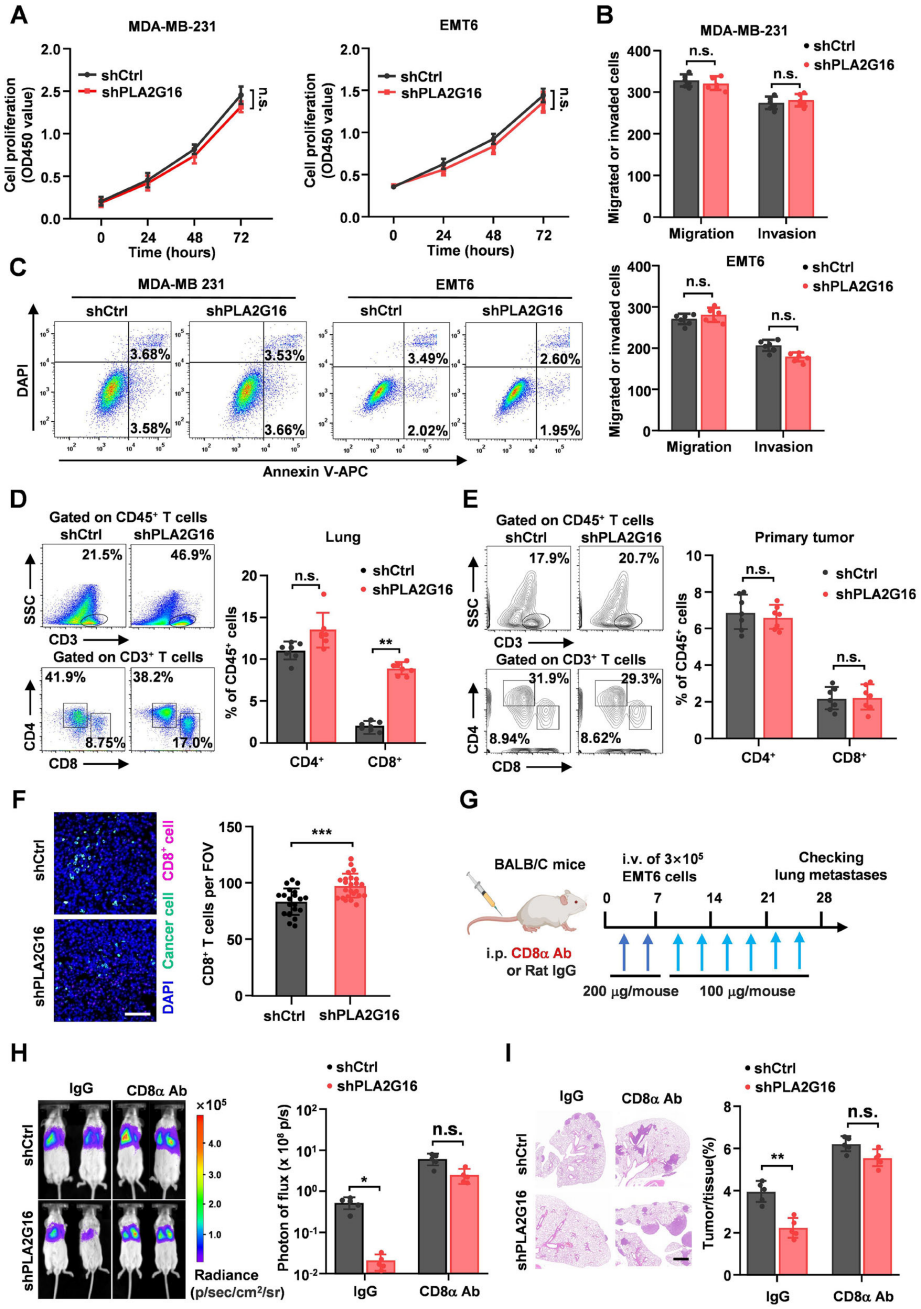
PLA2G16‐driven lung metastasis of breast tumor cells is related with lung infiltrating CD8^+^ T cell inactivation. A–C) In vitro analysis of tumor cell proliferation A), migration or invasion B) and apoptosis C) after PLA2G16 knockdown (*n* = 3 A), or *n* = 6 B)). D–E) Flow cytometry to determine the ratio of CD8^+^ T cells in CD45^+^ immunocytes isolated from the lung parenchyma at 72 h after mice were injected with PLA2G16 knockdown or parent EMT6 cells (D) (*n* = 7), or in primary breast tumor (E) (*n* = 7). F) IF shown CD8^+^ T cells accumulation in lungs at 72 h after intravenous injection of GFP^+^ EMT6 tumor cells (*n* = 24 random microscopic fields (RMFs) from 3 mice per group). G) Schematic illustration of CD8^+^ T cells depletion in tumor bearing mice by i.p. injection of CD8α antibodies or control IgG. The blue arrow indicates CD8^+^ T cells depletion by injecting CD8α antibodies (200 µg/mouse) for consecutive two times a week before tumor inoculation, while the sky‐blue arrow indicates the maintenance injection of CD8α antibodies (100 µg/mouse) twice a week for three weeks after cancer cell inoculation. H–I) Representative images of BLI (H, *n* = 5), and H&E images (I, *n* = 5) in lungs of mice treated with IgG or the CD8^+^ T cell clearance antibody at week 3 after tumor inoculation. Scale bars: 1 mm. Data are the mean ± SD. ^*^ *p* < 0.05, ^**^ *p* < 0.01, and ^***^ *p* < 0.001 by two‐tailed unpaired t test. n.s., not significant.

To investigate how tumoral PLA2G16 regulates lung metastasis, we explored the effects of PLA2G16 on intrinsic malignant characteristics of parental tumor cells and found that PLA2G16 knockdown (**Figure** **2**A–C) or ectopic PLA2G16 overexpression (Figure S4A–C, Supporting Information) had no significant effects on cell proliferation, migration, invasion and apoptosis of parental tumor cells in vitro. In contrast to these findings in vitro (Figure 2A,C; Figure S4A–C, Supporting Information) and the data acquired from mice (Figure 1E–J), we interestingly found that PLA2G16 could affect TNBC lung metastasis in immunocompetent BALB/c mice rather than in lymphocyte‐deficient BALB/c nude mice, strongly indicating that the pro‐metastatic role of PLA2G16 in TNBC is related with dysfunction of T lymphocyte in lung metastatic microenvironment.

**Figure 3:**
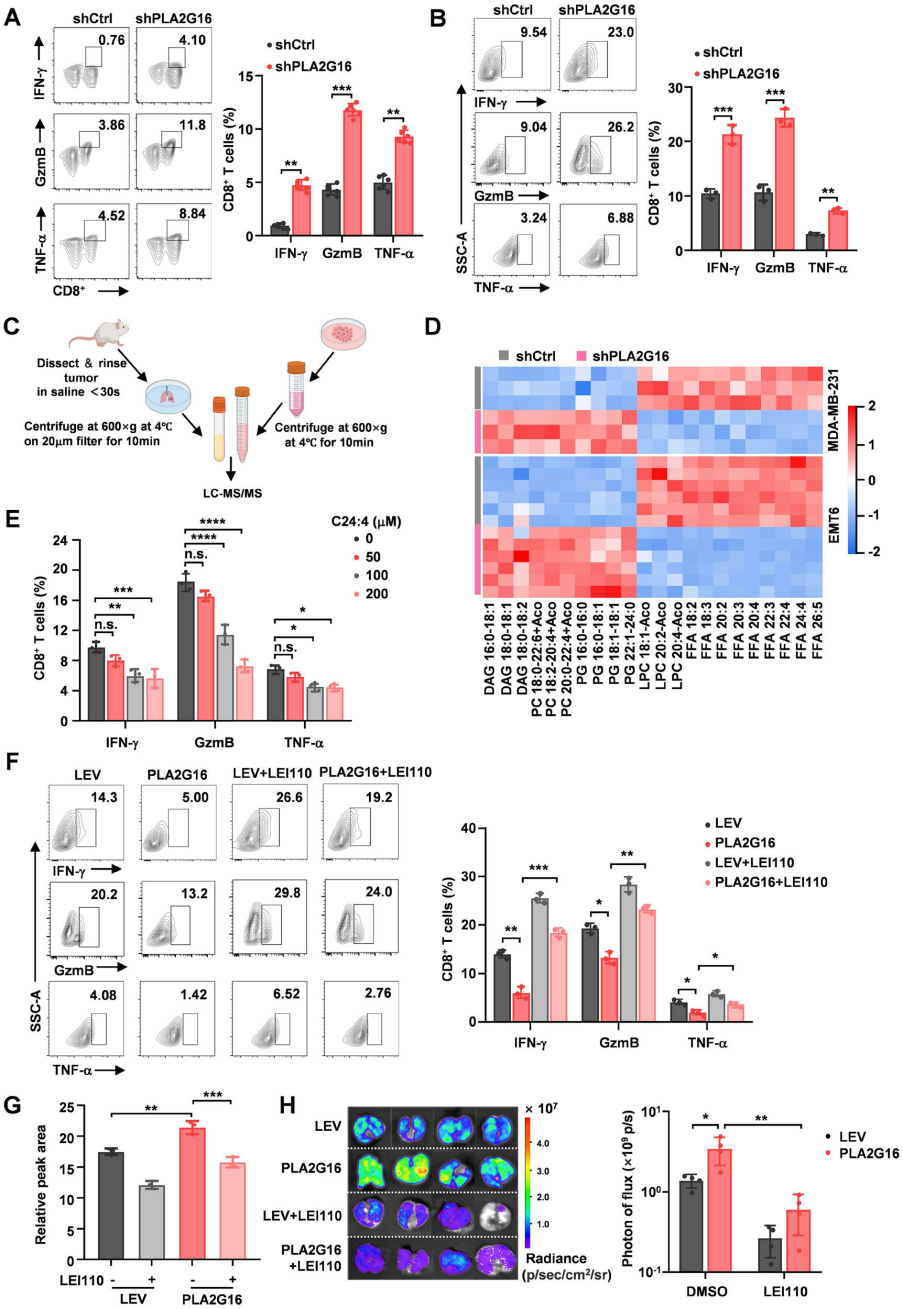
Tumoral PLA2G16 mediates the accumulation of lipid metabolite C24:4 (n‐6) in lung microenvironment to inhibit the activation and effector functions of CD8^+^ T cells in lung. A) The percentage of cytokines produced by CD8+ T cells in mouse lung was measured by FACS analysis (*n* = 6). B) Mouse naive CD8+ T cells were co‐cultured with PLA2G16 knockdown or control EMT6 cells, the cytokines production of CD8^+^ T cells were determined by FACS analysis (*n* = 3). C) Experiment scheme. D) Heatmap of 22 significantly changed metabolites (*p* < 0.05, unpaired two‐tailed Student's t‐test). Red indicates increase, and blue indicates decrease. −2–2 indicates the Fold Change. E) Mouse naive CD8+ T cells treated with or without C24:4 (n‐6), and cytokine productions were detected by FACS analysis (*n* = 3). F) Mouse naive CD8+ T cells co‐cultured with PLA2G16 overexpressed or control PY8119 cells under pre‐treatment with or without LEI110 (200 nm). The cytokine production from CD8+ T cells was determined by FACS analysis (*n* = 3). G) C24:4 (n‐6) levels were detected in the lung interstitial fluid of mice bearing PY8119 (LEV/PLA2G16) lung metastases following LEI110 treatment (5 mg kg^−1^, once every two days) for three weeks (*n* = 3). H) Representative images of BLI and quantification of lung metastases in mice treated with DMSO or LEI110 (*n* = 4). Data are the mean ± SD. ^*^ *p* < 0.05, ^**^ *p* < 0.01, and ^***^ *p* < 0.001 by two‐tailed unpaired *t* test. n.s., not significant.

Therefore, we tried to assess the changes of lymphocyte populations in the metastatic lung during the metastatic seeding stage. Performing flow cytometry analysis, we uncovered that loss of PLA2G16 in EMT6 tumor cells led to an increase (Figure 2D), while ectopic PLA2G16 overexpression in PY8119 tumor cells resulted in a reduction (Figure S4D) of CD45^+^CD3^+^CD8^+^ T cell population in TNBC lung metastatic microenvironment. There were negligible changes of CD45^+^CD3^+^CD8^+^ T cell abundance in the primary tumor (Figure 2E) and spleen of the tumor‐burden mice (Figure S4E Supporting Information), hinting that the reduction of CD8^+^ T cell population in metastatic lung was not attributed to their biogenesis alteration in immunocompetent mice. In addition, there was a negative correlation between the tumoral PLA2G16 level and the deficiency of CD8^+^ T cells in the metastatic seeding stage (Figure 2F). Furthermore, specifically depleting CD8^+^ T cells by anti‐CD8α clearance antibody in mice (Figure 2G; Figure S4F, Supporting Information), the PLA2G16‐knockdown induced survival disadvantage of DTCs could be reversed in the metastatic early stage (Figure S4G, Supporting Information), leading to more lung metastatic foci (Figure 2H,I). Compared with the pulmonary infiltrating CD8^+^ T cells from the mice injected with control EMT6 cells, the pulmonary infiltrating CD8^+^ T cells from the mice injected with PLA2G16‐deficient EMT6 cells exhibited significant functional activities, such as higher level of cytokines (IFN‐γ, TNF‐α, and granzyme B) (**Figure** **3**A) and CD44 expression (an activation marker of CD8^+^ T cells) (Figure S4H, Supporting Information). Co‐culturing these pulmonary infiltrating CD8^+^ T cells with parent EMT6 cells, the pulmonary CD8^+^ T cells isolated from mice with PLA2G16‐knockdown EMT6 metastases had stronger anti‐tumor immunity (Figure S4I, Supporting Information). Then mouse naive CD8^+^ T cells were co‐cultured with PLA2G16 knockdown or control EMT6 cells, and the CD8^+^ T cell activities were assessed (Figure S5A). In accordance with in vivo findings (**Figure** 3A), PLA2G16 knockdown in tumor cells resulted in activated CD8^+^ T cells, which had significantly higher cytokines (Figure 3B). Strikingly, naive CD8^+^ T cells co‐cultured with PLA2G16‐silenced EMT6 cells had a notably decreased PD‐1 and TIM3 expression, the well‐known markers of CD8^+^ T cell exhaustion (Figure S5B, Supporting Information). Taken together, these data demonstrate that PLA2G16 may serve as a functional facilitator for TNBC lung metastasis through inhibiting CD8^+^ T cell activation.

### 2.5 Tumoral PLA2G16‐Induced Accumulation of Fatty Acid C24:4 (n‐6) in Lung Microenvironment Plays an Essential Role in CD8^+^ T Cell Inactivity

Next, we interrogated how PLA2G16 affects the activation of CD8^+^ T cells infiltrating in lung. It has been reported that PLA2G16 can catalyze phosphatidic acid into free fatty acids (FFAs),^[^ ^26^ ^]^ and LC‐PUFAs act as crucial modulators to T cell proliferation, activation, and immune function.^[^ ^21^, ^36^ ^]^ To better understand the potential mechanism underlying the suppression of CD8^+^ T cells in the lung metastatic microenvironment of PLA2G16^high^ TNBC, we first profiled the content and abundance of various lipids within conditioned medium of MDA‐MB‐231 cell culture and lung interstitial fluid, which refers to the “‘extracellular milieu”’ of the lung metastatic tumor microenvironment (TME),^[^ ^37^, ^38^, ^39^ ^]^ from mice with intravenous injection of EMT6 cells by LC‐MS/MS analysis (Figure 3C). There were 317 and 138 altered metabolites in MDA‐MB‐231 cell‐conditioned medium (Figure S5C, Supporting Information) and lung interstitial fluid from EMT6 injected mice (Figure S5D, Supporting Information), respectively. Among these metabolites, 22 significantly altered metabolites existed in both MDA‐MB‐231 cell conditioned medium and lung interstitial fluid from EMT6 lung metastasis mice (Figure 3D), and nine of them were free FFAs (Figure S5E, Supporting Information). Checking the concentrations of these FFAs in the primary tumor interstitial fluid and lung interstitial fluid, we found that there was a higher level of long‐chain polyunsaturated fatty acids (C20:4, C22:4, and C24:4 (n‐6)) in lung interstitial fluid than in primary tumor interstitial fluid (Figure S5F, Supporting Information). Based on the quantitative results of LC‐MS/MS analysis, we set up a reasonable fatty acid concentration gradient of 0, 50, 100, and 200 µm to assess the effects of the three LC‐PUFAs on CD8^+^ T cell activity in vitro. Treating CD8^+^ T cells with C20:4, C22:4 and C24:4 (n‐6), we found that C24:4 (n‐6) (Figure 3E; Figure S5G, Supporting Information), rather than the other two LC‐PUFAs, induced a marked reduction of cytokines and an elevated PD‐1 and TIM3 in CD8^+^ T cells (Figure S5H–J, Supporting Information).

To identify the primary source of C24:4 (n‐6) in the lung microenvironment, we first measured *Pla2g16* mRNA levels across different lung cell types in mice bearing EMT6 (shCtrl/shPLA2G16) metastatic tumors. We found that DTCs exhibited the highest PLA2G16 expression, and importantly, knockdown of PLA2G16 in tumor cells significantly reduced C24:4 (n‐6) levels in the lung interstitial fluid (Figure S5K,L, Supporting Information), indicating that DTCs contribute to the accumulation of C24:4 (n‐6) in lung interstitial fluid. To further confirm whether C24:4 (n‐6) is related with PLA2G16 and inactivation of CD8^+^ T cells, we measured C24:4 (n‐6) contents in the conditioned medium of ectopic PLA2G16 engineering PY8119 (PY8119/PLA2G16) cells or cells with or without LEI110 (an inhibitor of PLA2G16) treatment, and found that C24:4 (n‐6) was regulated by PLA2G16 (Figure S5M,N, Supporting Information). Co‐culturing CD8^+^ T cells with ectopic PLA2G16 engineering PY8119 cells (PY8119/PLA2G16) or LEI110‐treated PY8119/PLA2G16 cells, PLA2G16^high^ tumor cells significantly inhibited the cytokine levels in CD8^+^ T cells and increased PD1^+^TIM3^+^ CD8^+^ T cell population, which could be abolished by LEI110 treatment (Figure 3F; Figure S5O, Supporting Information). Meanwhile, LEI110 treatment reduced C24:4 (n‐6) levels in the lung interstitial fluid, and the pro‐metastatic effects of PLA2G16 were significantly attenuated by LEI110 treatment in vivo (Figure 3G,H). Collectively, these data demonstrate that C24:4 (n‐6) accumulation in TNBC metastasis lung modulates CD8^+^ T cell activation and immune response.

### 2.6 Fatty Acid C24:4 (n‐6)‐Mediated PPARα Signaling Activation Triggers CD8^+^ T Cell Dysfunction

**Figure 4:**
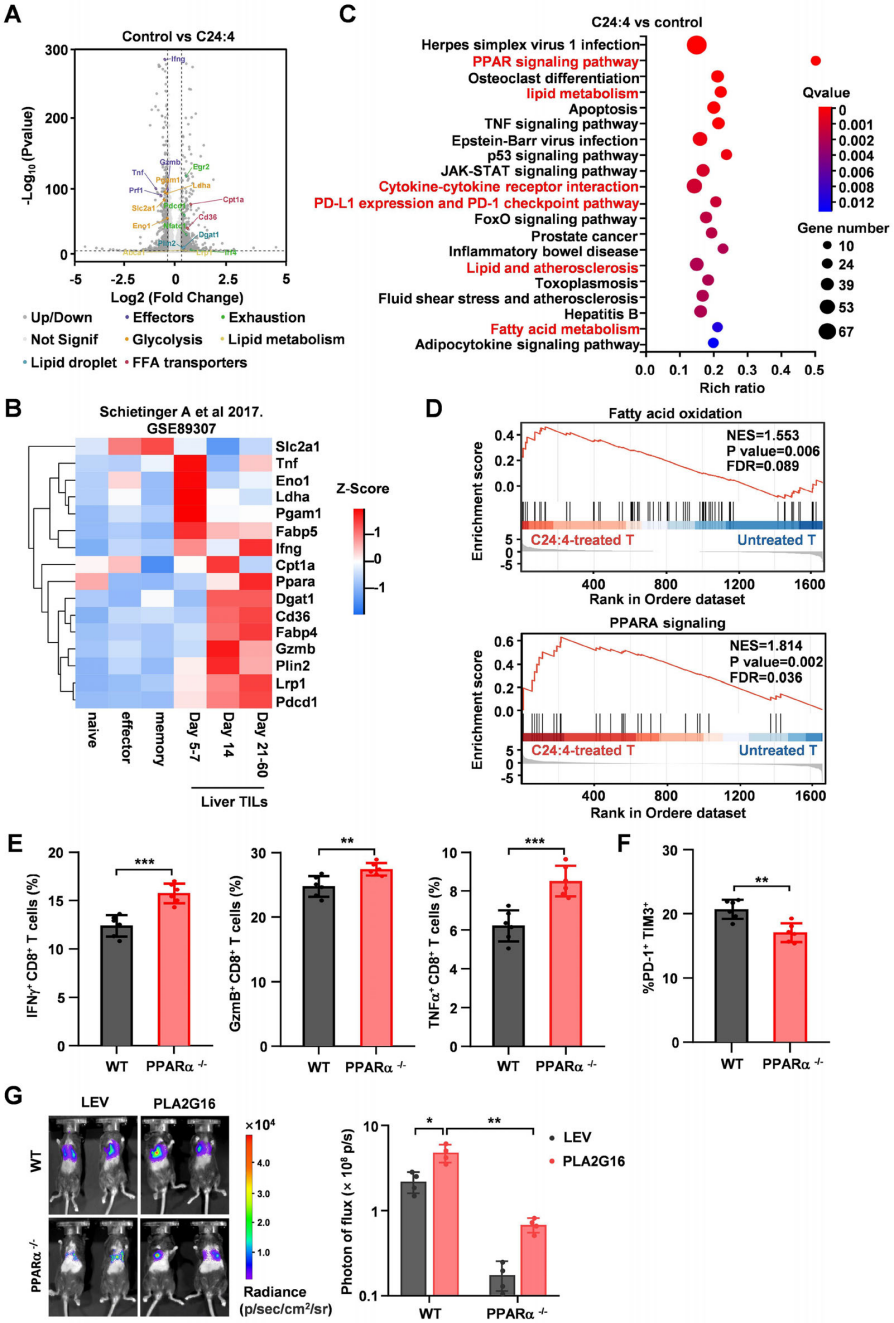
Lipid metabolite C24:4 (n‐6) triggers CD8^+^ T cell dysfunction by PPARα signaling. A) Volcano plot showing the differentially expressed genes between control and C24:4 (n‐6) treated mouse naive CD8^+^ T cells. The significantly differential expression genes related to lipid, glycolysis and effectors are highlighted (Fold change>1.5 and false discovery rate (FDR) < 0.05). B) The expression levels of indicated genes (e.g., *Cd36, Lrp1, Plin2* and *Ppara*) in naive, effector, memory TCRTAG CD8^+^ T cells were analyzed using the RNAseq dataset GSE89307 (Schietinger et al., 2017). Heatmap shows the mRNA levels normalized in row Z‐score. C) Pathways enrichment analysis of KEGG in C24:4 (n‐6)‐treated CD8^+^ T cells compared with control CD8^+^ T cells. D) GSEA enrichment plots of changed fatty acid oxidation and PPARα targets in C24:4 (n‐6)‐treated CD8^+^ T cells compared with control CD8^+^ T cells. Red represents a high expression level and blue indicates a low expression level. E,F) In WT or *Pparα^−/−^* mice injected with ectopic PLA2G16 or control PY8119 tumor cells, cytokine production (E, *n* = 6) and percentage of terminally exhausted T cells (PD‐1^+^ TIM‐3^+^) in total CD8^+^ T cells from lung metastases (F, *n* = 6) were determined by FACS analysis. G) Intravenous injection of ectopic PLA2G16 or control PY8119 tumor cells for lung colonization analysis. Representative images of BLI in each group (left panel) and BLI quantification of the experimental groups (*n* = 4, right panel) are shown. Data are the mean ± SD. ^*^ *p* < 0.05, ^**^ *p* < 0.01, and ^***^ *p* < 0.001 by two‐tailed unpaired *t* test. n.s., not significant.

To explore why C24:4 (n‐6) triggers immune dysfunction of CD8^+^ T cells, RNA sequencing (RNA‐seq) was performed to check the expression profile between C24:4 (n‐6) treated and untreated CD8^+^ T cells. There were 1648 differentially expressed genes (DEGs) in C24:4 (n‐6) treated CD8^+^ T cells compared with control CD8^+^ T cells, and the significantly altered genes were shown in Figure S6A (Supporting Information). Among them, genes encoding cytokines (e.g., *Prf1, Tnf*, and *Gzmb*) were notably downregulated, while genes encoding exhaustion markers (e.g., *Pdcd1, Egr2, Nfatc1*, and *Irf4*) were remarkably upregulated and enriched in C24:4 (n‐6) treated CD8^+^ T cells (**Figure** **4**A; Figure S6B, Supporting Information). What's more, many genes involved in lipid transporting (*Cd36* and *Cpt1a*), lipid‐droplet formation and lipases (*Dgat2* and *Plin2*), and lipid metabolism (*Abca1* and *Lrp1*) were up‐expressed in C24:4 (n‐6) treated CD8^+^ T cells, indicating a lipid metabolism‐related activation in these CD8^+^ T cells (Figure 4A). A similar phenotype existed in CD8^+^ TILs from SV40 T antigen (TAG)‐driven murine liver tumors (GSE89307), that is, the transcripts of lipid metabolism associated genes, as mentioned above, were gradually upregulated as CD8^+^ TILs were progressively becoming exhausted (Figure 4B). By KEGG pathway analysis, metabolic pathways associated with lipid metabolism, PD‐1/PD‐L1 signals, and PPAR signaling pathway were obviously enriched in C24:4 (n‐6) treated CD8^+^ T cells (Figure 4C). As ligand‐activated transcription factors, PPARs (including PPARα, β/δ, γ) can be activated by fatty acids or their derivatives and serve as a bridge between disorders of lipid metabolism and innate immunity; PPARs‐regulated genes, such as genes encoding lipogenic or lipolytic enzymes, were involved in lipid metabolism.^[^ ^39^, ^40^ ^]^ Intriguingly, there was a set of enriched genes associated with fatty acid oxidation (FAO) and PPARα targets in C24:4 (n‐6)‐treated CD8^+^ T cells compared with the untreated group (Figure 4D). Moreover, C24:4 (n‐6) dose‐dependently increased the protein expression of PPARα targets, such as CPT1A, DGAT1, CD36, and FABP1 proteins, in CD8^+^ T cells (Figure S6C, Supporting Information), indicating that PPARα‐regulated genes potentially involve in FAO of CD8^+^ T cells.

We thus asked whether PPARα is crucial for C24:4 (n‐6)‐induced CD8^+^ T cell dysfunction. GW6471, a PPARα inhibitor, significantly attenuated C24:4 (n‐6)‐mediated suppressive effects on CD8^+^ T cell activities, resulting in enhanced cytokine production (Figure S6D, Supporting Information), reduced PD‐1^+^TIM3^+^CD8^+^ T cells (Figure S6E, Supporting Information), and increased CD44^+^ CD8^+^ T cell population (Figure S6F, Supporting Information). Whereas PPARβ/δ inhibitor GSK0660 (Figure S6G,H, Supporting Information) or PPARγ inhibitor GSK3787 (Figure S6I,J, Supporting Information) had no effects on CD8^+^ T cell immune functions. Blockage of PPARα signaling, potentially restoring CD8^+^ T cell immune function, prompted us to investigate whether PPARα knockout (KO) could promote the anti‐tumor immunity of CD8^+^ T cells in vivo and inhibit lung metastasis of breast cancer. CD8^+^ T cells from PPARα^−/−^ mice had higher cytokines (Figure 4E) and lower exhaustion profile expression (Figure 4F) compared to CD8^+^ T cells from the wild type (WT) litter‐mates. Meanwhile, PPARα deficiency abolished the pro‐metastatic effects of the enhanced PLA2G16 expression in vivo, similar to those of LEI110 targeting PLA2G16 (Figure 4G). These findings demonstrate that PPARα is a key regulator in C24:4 (n‐6)‐induced CD8^+^ T cell immune dysfunction.

### 2.7 C24:4 (n‐6)‐PPARα Axis Mediates CD8^+^ T Cell Dysfunction by Promoting FAO While Reducing Glycolysis

Actually, metabolic reprogramming plays a critical role in regulating T cell functions and their adaptation to a complex microenvironment.^[^ ^41^ ^]^ CD8^+^ T effector (T_EFF_) cells depend on aerobic glycolysis to maintain their antitumor effector function, whereas FAO may be essential for CD8^+^ T cell longevity.^[^ ^42^ ^]^ PPARα, a fatty acid‐activated transcription factor, is the key regulator for FAO by triggering the transcription of genes involved in FAO.^[^ ^43^ ^]^

**Figure 5:**
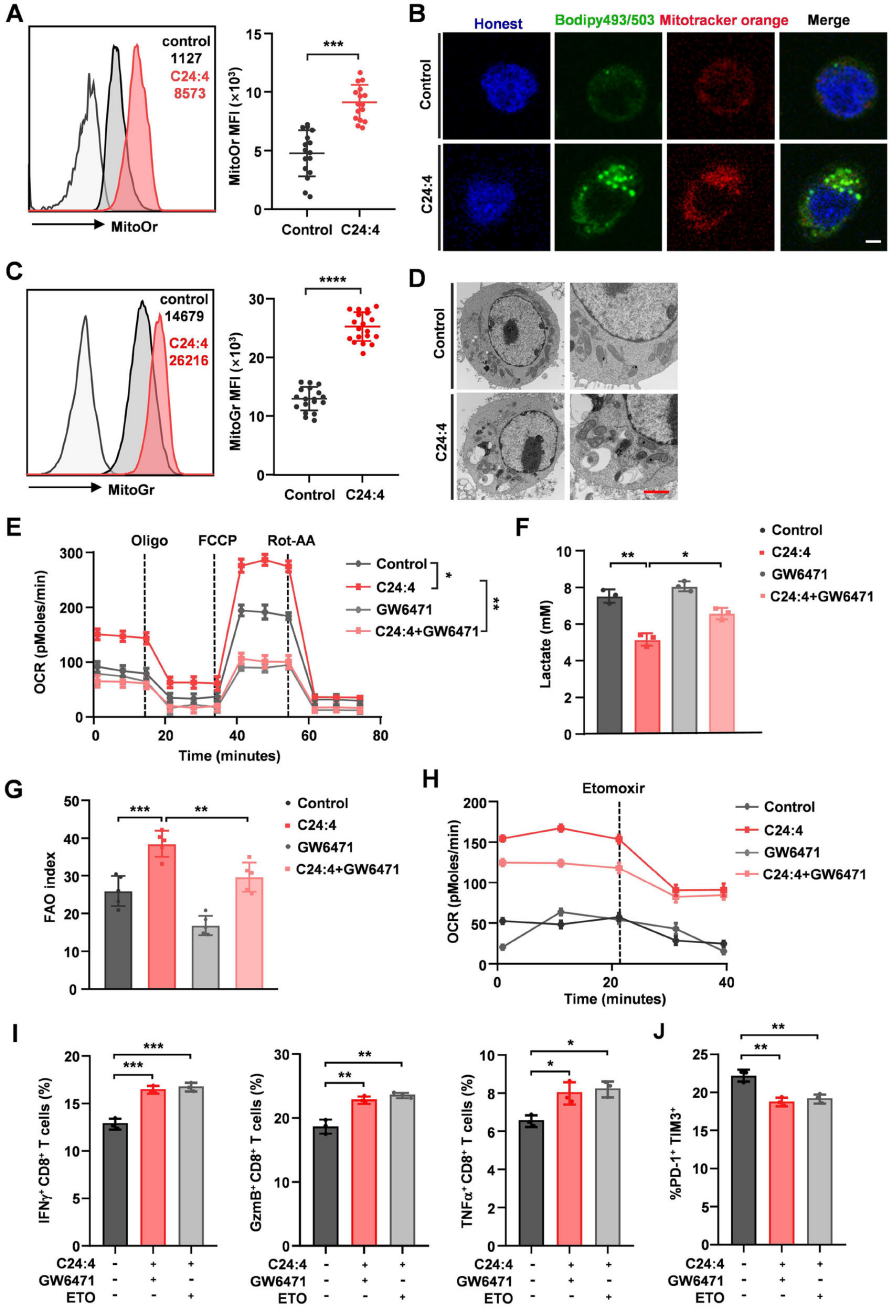
PPARα activation regulates fatty acid oxidation and glycolysis metabolism of CD8^+^ T cells. A) Representative histogram (left) and quantitative MFI (numbers in the image on the left) of Mitotracker orange staining in CD8^+^ T cells (*n* = 16). B) Localization of the incorporated lipids with Mitotracker orange in CD8^+^ T cells (Scale bar, 10 µm). C) Representative histogram (left) and quantitative MFI (numbers in the image on the left) of Mitotracker green staining in CD8^+^ T cells (*n* = 18). D) Representative image of transmission electron microscope (TEM) on control and C24:4 (n‐6) conditioned mouse naive CD8^+^ T cells (Scale bar, 500 nm). E,F) After CD8^+^ T cells were pre‐treated with or without C24:4 (n‐6) (100 µm) in the presence or absence of GW6471 (15 µm) for 48 h, the OCR (E, *n* = 3), lactate production (F, *n* = 3) and FAO activity (F, *n* = 5) in CD8^+^ T cells were measured. G) CD8^+^ T cells pretreated with or without C24:4 (n‐6) (100 µm) in the presence or absence of GW6471 (15 µm) for 48 h, FAO was evaluated (*n* = 3). H) CD8^+^ T cells were incubated with or without C24:4 (n‐6) (100 µm) in the presence or absence of GW6471 (15 µm) or ETO (40 µm). The amount of OCR derived from FAO was quantified as the reactivity of CD8^+^ T cells in response to ETO treatment (*n* = 5). I,J) The indicated cytokine production (I) (*n* = 3) and PD1 and TIM3 levels (J, *n* = 3) were assessed by FACS analysis. Data are the mean ± SD. ^*^ *p* < 0.05, ^**^ *p* < 0.01, and ^***^ *p* < 0.001 by two‐way ANOVA in (E) and (H) and two‐tailed unpaired *t* test in (others). n.s., not significant.

To decode whether C24:4 (n‐6)‐mediated activation of PPARα signaling acts as a crucial contributor in regulating CD8^+^ T cell metabolic reprogramming, we first assessed FAO‐related mitochondrial content and function in C24:4 (n‐6) administrated CD8^+^ T cells and control CD8^+^ T cells. Naturally, the mitochondrial membrane potential of CD8^+^ T cells notably increased after exposure to C24:4 (n‐6) (**Figure** **5**A,B; Figure S7A, Supporting Information). The increased mitochondrial function by C24:4 (n‐6) was accompanied by an augmented mitochondria quantity (Figure 5C,D; Figure S7B, Supporting Information), suggesting that more mitochondria and higher mitochondrial capability are required for C24:4 (n‐6)‐treated CD8^+^ T cells to deal with increased FAA metabolism. Measuring Oxygen consumption rate (OCR) (an index of oxidative phosphorylation) (Figure 5E; Figure S7C, Supporting Information), Extracellular Acidification Rate (ECAR) (an index of glycolytic ability) (Figure S7D,E, Supporting Information) and lactate products (Figure 5F) of CD8^+^ T cells, we observed a robust Oxidative phosphorylation FAO Fatty acid oxidation (OXPHOS) and reduced glycolysis in C24:4 (n‐6)‐treated CD8^+^ T cells, which could be reversed by GW6471 treatment. Further findings from direct measurement of FAO potential revealed that C24:4 (n‐6) could promote FAO activity of CD8^+^ T cells, which was abrogated by GW6471 (Figure 5G). ETO, an inhibitor of FAO, could overrule C24:4 (n‐6)‐PPARα advantages on OCR (Figure 5H) and restored the functional activities of C24:4 (n‐6)‐treated CD8^+^ T cells, as GW6471 did (Figure 5I,J). To demonstrate whether elevated FAO impairs CD8⁺T cell function in vivo, we treated mice injected PY8119 (LEV/PLA2G16) with ETO. ETO treatment effectively reduced FAO levels, restored cytokine production, and ultimately led to a significant reduction in lung metastatic burden in mice (Figure S7F–H, Supporting Information). Taken together, these data demonstrate that C24:4 (n‐6) is involved in lipid metabolic reprogramming in dysfunctional CD8^+^ T cells and PPARα is essential for C24:4 (n‐6)‐induced immune dysfunction of CD8^+^ T cells.

### 2.8 C24:4 (n‐6) Promotes FAO in CD8^+^ T Cells by Modulating Nuclear Import of PPARα

**Figure 6:**
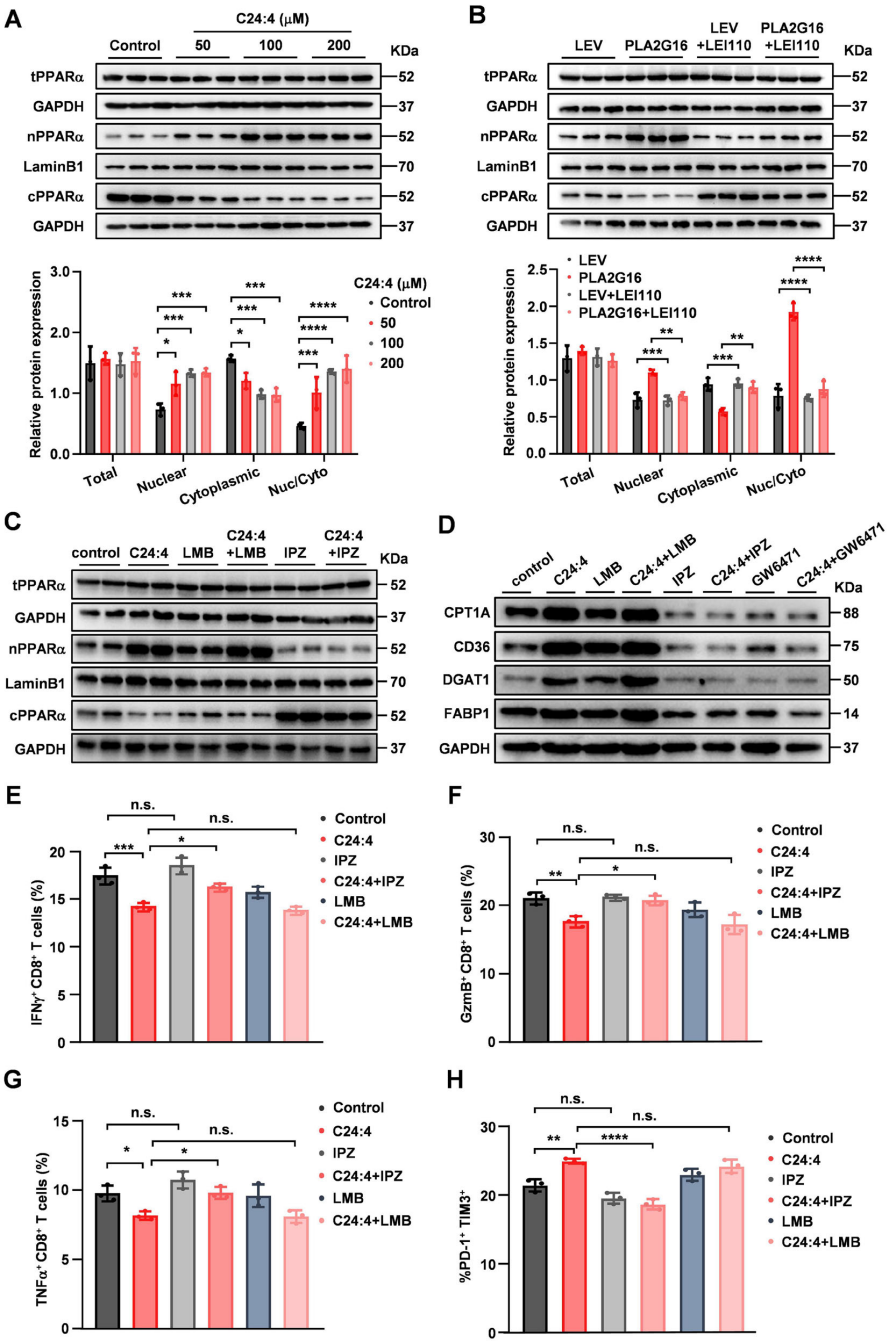
C24:4 (n‐6) stimulates nuclear accumulation of PPARα and promotes the expression of PPARα target genes. A) After mouse naive CD8^+^ T cells treated with or without C24:4 (n‐6), PPARα levels were evaluated by western blotting. B) C57BL/6J mice were intravenously injected with ectopic PLA2G16 overexpressed or control PY8119 cells and administered with or without LEI110 (5 mg kg^−1^, once every two days) for three weeks. PPARα expression levels were assessed in CD8^+^ T cells from lung parenchyma by western blotting. C,D) Mouse naive CD8^+^ T cells treated with Importazole (IPZ, 10 µm), Leptomycin B (LMB, 10 ng µL^−1^), and/or C24:4 (n‐6). PPARα levels C) and the PPARα target proteins D), such as CPT1a, DGAT1, CD36, and FABP1, were evaluated by western blotting. E–H) Mouse naive CD8^+^ T cells treated with Importazole (IPZ, 10 µm), Leptomycin B (LMB, 10 ng µL^−1^), and/or C24:4 (n‐6). The indicated cytokine production, IFNγ (E, *n* = 3), GzmB (F, *n* = 3), TNFα (G, *n* = 3), and PD‐1 expression (H, *n* = 3) were assessed by FACS analysis. Data are the mean ± SD. ^*^ *p* < 0.05, ^**^ *p* < 0.01, and ^***^ *p* < 0.001 by two‐tailed unpaired t test. n.s., not significant.

Highly polyunsaturated FFAs act as natural ligands to PPARα activation in triggering the transcription of target genes.^[^ ^44^, ^45^ ^]^ By analyzing PPARα levels and their location within CD8^+^ T cells, we found that C24:4 (n‐6) could dose‐dependently increase the nuclear PPARα proteins, but not affect total PPARα levels in CD8^+^ T cells (**Figure** **6**A). Consistently, the CD8^+^ T cells isolated from metastatic lungs of mice injected with ectopic PLA2G16 expressing PY8119 cells at 72 h also displayed more nuclear PPARα proteins and higher nuclear to cytoplasmic ratio of PPARα than the CD8^+^ T cells from lung of control mice, which could be abolished by LEI110 treatment (Figure 6B), suggesting C24:4 (n‐6) facilitates nuclear localization of PPARα.

To understand the accumulation of nuclear PPARα being tightly linked with FAO‐associated gene expression, we treated CD8^+^ T cells with leptomycin B (LMB), an inhibitor of nuclear export receptor, and importazole (IPZ), an inhibitor of nuclear import receptor in combination with C24:4 (n‐6). As expected, C24:4 (n‐6)‐induced PPARα accumulation in the nucleus could be remarkably mitigated by IPZ, and obviously increased by LMB treatment (Figure 6C). Correspondingly, C24:4 (n‐6)‐stimulated PPARα target proteins, such as CPT1a, DGAT1, CD36, and FABP1, were significantly suppressed under IPZ treatment and slightly increased by LMB (Figure 6D); meanwhile, inhibiting PPARα activity by GW6471 almost abrogated C24:4 (n‐6)‐induced upregulation of these target proteins (Figure 6D). Furthermore, accumulation of nuclear PPARα obviously steered the anti‐tumor activity of CD8^+^ T cells to immune dysfunction, and blockage of PPARα location in the nuclear by IPZ restored the anti‐tumor activity of CD8^+^ T cells (Figure 6E–H). Therefore, C24:4 (n‐6) regulates PPARα accumulation in the nucleus by promoting nuclear import of PPARα to promote FAO and modulate inactivation of CD8^+^ T cells in the lung metastatic microenvironment.

### 2.9 Targeting PLA2G16 Improves the Therapeutic Effect of Anti‐PD‐1‐Based Immunotherapy

**Figure 7:**
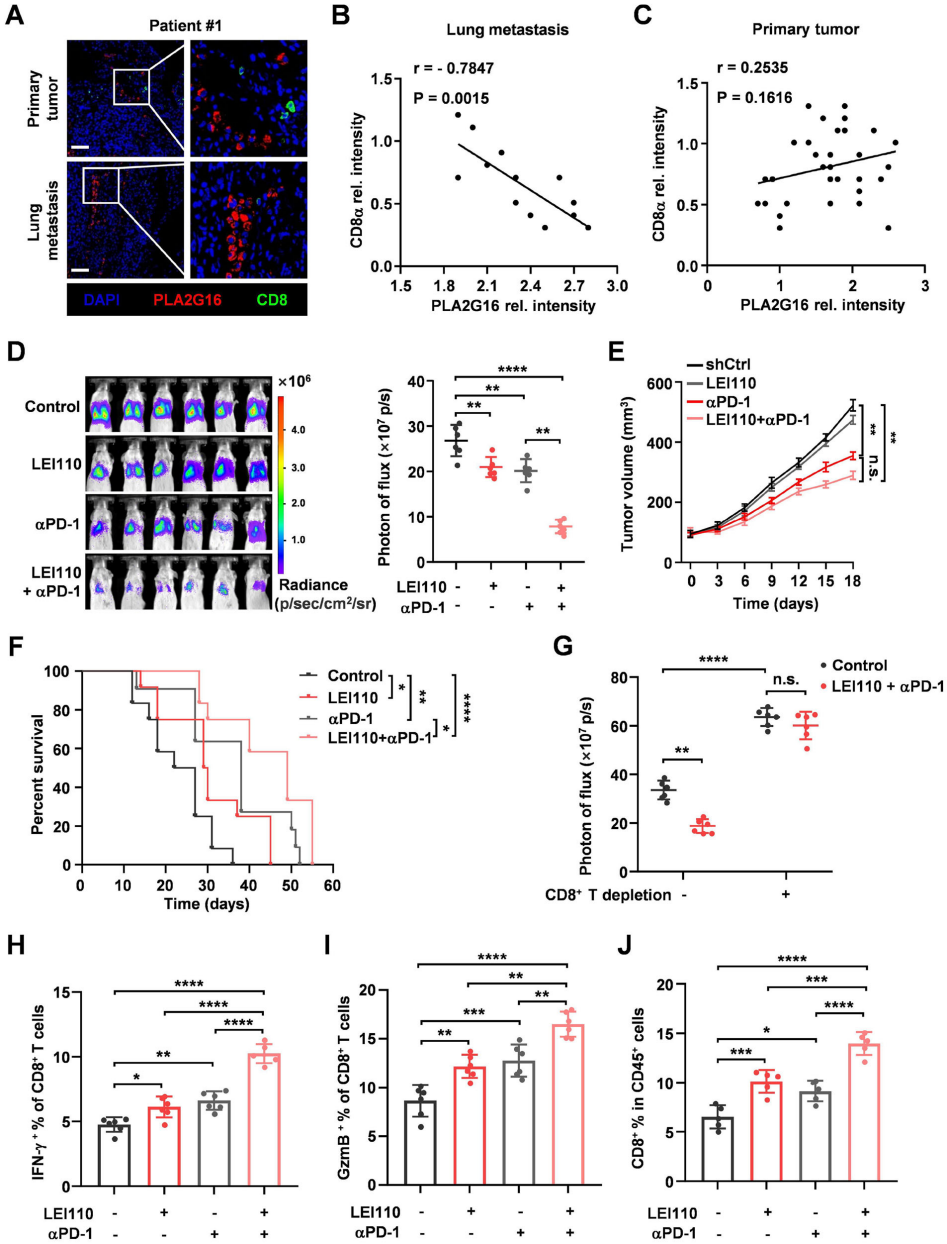
Pharmacological inhibition of PLA2G16 mitigates lung metastasis and improves the therapeutic effect of anti‐PD‐1‐based immunotherapy in mouse models. A–C) IF analyses of PLA2G16 levels and CD8^+^ T cell numbers in primary breast tumors (*n* = 32) and lung metastases (*n* = 13). Shown are representative images A) and correlation of PLA2G16 expression to CD8^+^ T cell infiltration in lung metastasis B) and primary tumor C). D) The combined effects of anti‐PD‐1 and LEI110 on lung metastasis of BALB/c mice bearing EMT6 tumor were determined by BLI. Shown are the representative images of BLI (left panel) and quantification (*n* = 6, left panel). E) EMT6 tumor growth curve of BALB/c mice with indicated treatments. F) Kaplan–Meier survival curve for EMT6‐bearing mice. Control, *n* = 12; anti‐PD‐1, *n* = 11; LEI110, *n* = 12; LEI110 + anti‐PD‐1, *n* = 12. G) Lung metastases of CD8^+^ T‐depleted mice (*n* = 6 for control and LEI110 + anti‐PD‐1) and normal mice (*n* = 6 for control and LEI110 + anti‐PD‐1) were determined by BLI. H–J) IFN‐γ (H) and GzmB (I) production of CD8^+^ T cells (*n* = 6) and percentage of CD8^+^ T cells in mice lung J) (*n* = 5) were measured by FACS analysis after the indicated treatment. Data are the mean ± SD. ^*^ *p* < 0.05, ^**^ *p* < 0.01, and ^***^ *p* < 0.001 by Pearson's correlation analysis in (B) and (C), log rank test in (E), two‐way ANOVA in (F), and two‐tailed unpaired *t* test in others. n.s., not significant.

In order to consolidate the therapeutic potential of PLA2G16, we first evaluated the clinical relevance of tumoral PLA2G16 and CD8^+^ T cells infiltration in human TNBC. There was an obvious negative correlation of PLA2G16 level and CD8^+^ T cells infiltration in TNBC lung metastases, which was more pronounced than in primary tumors (**Figure** **7**A–C; Figure S8A, Supporting Information). Next, we wondered whether the inactivation of human CD8^+^ T cells could also be caused by tumoral PLA2G16 expression or C24:4 (n‐6) treatment, as observed in mouse CD8^+^ T cells. Co‐culturing human CD8^+^ T cells from healthy donors with MDA‐MB‐231 cells or PLA2G16‐knockdown MDA‐MB‐231 cells in the presence or absence of C24:4 (n‐6), we discovered that human CD8^+^ T cells under C24:4 (n‐6) treatment or co‐culturing with PLA2G16^high^ tumor resulted in reductions of cytokines but increases of TIM‐3 and PD‐1 (Figure S8B–E, Supporting Information). LEI110 and GW6471 treatment restored human CD8^+^ T cell activation (Figure S8F–I, Supporting Information).

Accumulating studies support that T cell exhaustion in tumors contributes to immunotherapy resistance, and growing studies are being conducted to investigate strategies for converting immune “cold” tumors into “hot” tumors to reach higher ICB responsiveness.^[^ ^46^ ^]^ In order to explore the potential for PLA2G16 inhibition to augment the therapeutic efficacy of PD‐1 blockage, EMT6 tumor‐burden mice were treated with anti‐PD‐1 antibody, LEI110, or LEI110 combined with anti‐PD‐1, respectively. In comparison to LEI110 or anti‐PD‐1 antibody treatment alone in the EMT6 mouse model, the combined treatment of LEI110 and anti‐PD‐1 antibody showed the strongest therapeutic response in lung metastasis and significantly impaired tumor growth (Figure 7D,E), which further led to substantial survival advantages (Figure 7F). Moreover, the effectiveness of combination therapy was nearly eliminated after depletion of CD8^+^ T cells in mice (Figure 7G). Moreover, the combination treatment resulted in increased cytokines production (IFN‐γ and granzyme B) in CD8^+^ T cells from lung metastases (Figure 7H,I), as well as a general increase of infiltrating CD8^+^ T cells in the lung (Figure 7J). These findings demonstrate that the proposed combination treatments have a promising potential to improve clinical outcomes for metastatic TNBC patients by reinvigorating CD8^+^ T cell immune function and sensitizing PD‐1 blockade.

## 3 Discussion

Cancer cells significantly contribute to intertumoral heterogeneity in immunological contexts due to their complex metabolic profile and paracrine and juxtracrine communication.^[^ ^47^, ^48^ ^]^ Shedding light on tumor cell‐immune cell interaction in cancer progression and metastasis will enable the development of immunomodulatory strategies that are more tailored to patients with cancer. Here, we report that breast tumor cells with a higher level of PLA2G16 expression are more likely to survive in the lung and form metastases. Tumoral PLA2G16‐mediated accumulation of tetracosatetraenoic acid (C24:4 (n‐6)) in the lung interstitial fluid contributes to immune dysfunction of CD8^+^ T cells and impairs immune responses against malignant cells. Mechanistically, the uptake of C24:4 (n‐6) by CD8^+^ T cells stimulates PPARα signaling, which further induces LDs accumulation and shifts the metabolism of CD8^+^ T cells from a glycolytic state toward FAO. Crucially, targeting PLA2G16 effectively can correct the immune dysfunction of CD8^+^ T cells and improve the efficacy of clinical tumor immunotherapies.

As a class of cells with a dual role in the TME, immune cells exert both pro‐ and anti‐tumorigenic functions, and there is evidence that immune suppression and evasion are linked to cancer metabolism.^[^ ^49^, ^50^ ^]^ Much attention has recently been paid to which metabolites derived from cancer cells mediate the regulation of immune cell recruitment and function during cancer progression and metastasis.^[^ ^19^, ^51^, ^52^ ^]^ A variety of identified substances have varying effects on T cell activity in diverse ways. According to a recent study, loss of FH expression in tumors results in fumarate accumulation in tumor interstitial fluid and suppression of the activation and antitumor response in tumor‐infiltrating CD8^+^ T cells.^[^ ^51^ ^]^ Moreover, lactate, secreted from cancer cells during aerobic glycolysis, accumulates in the TME and can be utilized by immune cells, such as Treg cells, to drive proliferation and maintain their immunosuppressive function.^[^ ^52^ ^]^ However, these studies have focused on the primary sites of various tumors, with less attention being paid to the site of metastasis. Here, we observed that C24:4 (n‐6) derived from breast tumor cells with a high expression level of PLA2G16 shapes a lipid‐enriched lung metastasis microenvironment. Consistently, another recent study confirmed abundant fatty acid accumulation in lung interstitial fluid.^[^ ^53^ ^]^ Several other studies have also shown that LC‐FAs are emerging as an important but controversial class of metabolites involved in intercellular communication.^[^ ^20^, ^21^ ^]^ Therefore, it may be important for clinical cancer therapies to clarify whether all LC‐FAs have the potential to regulate CD8^+^ T cell immune response and the specific mechanism by which various LC‐FAs regulate different CD8^+^ T cell fate. Interestingly, among these LC‐FAs altered by PLA2G16 in our study, only C24:4 (n‐6) serves as a mediator in the crosstalk between cancer cells and immune cells, especially involving in CD8^+^ T cell dysfunction. The apparent biological difference among C24:4(n‐6), C20:4, and C22:4 prompted us to think about potential structural determinants underlying this specificity. The ligand‐binding domain of PPARα is notably large and promiscuous, allowing it to accommodate a variety of fatty acids, but the efficiency of activation is highly dependent on ligand properties.^[^ ^54^, ^55^ ^]^ The extended 24‐carbon chain and four double bonds of C24:4 (n‐6) may endow C24:4 (n‐6) with greater lipophilicity and a distinct 3D conformation compared to its shorter‐chain counterparts. These properties are likely to influence several critical parameters: 1) Receptor Affinity and Stability. The longer chain may form more extensive and stable van der Waals interactions within the spacious PPARα ligand‐binding pocket, potentially resulting in a higher binding affinity or a more stabilized active conformation of the receptor. 2) Cellular Uptake and Trafficking. Increased lipophilicity could facilitate more efficient diffusion or protein‐mediated transport across the plasma membrane, leading to higher intracellular concentrations in T cells. 3) Metabolic Stability. C24:4 (n‐6) may be less susceptible to rapid metabolic conversion or degradation than the well‐established substrate arachidonic acid (C20:4), thereby prolonging its half‐life and sustained signaling output. Future studies are warranted to directly test these hypotheses by quantifying binding constants, uptake kinetics, and the metabolic fate of these lipids. Nonetheless, our work highlights that subtle variations in lipid chain length may profoundly impact their immunoregulatory function, revealing a new layer of complexity in lipid‐mediated signaling within the TME.

It has been underscored in recent findings that metabolic reprograms are contributors to T cell survival, effector function, and differentiation.^[^ ^56^, ^57^, ^58^, ^59^ ^]^ As a case, the enhanced FAO, a hallmark of metabolic reprogramming, is necessary for CD8^+^ memory T cells, while glycolysis is critical for CD8^+^ effector T cells.^[^ ^60^ ^]^ CD8^+^ T cells with impaired immune function, such as anergic T cells and exhausted T cells, exhibit decreased glycolytic and/or oxidative metabolism.^[^ ^61^, ^62^ ^]^ However, there are studies suggesting that increased FAO by PPARs can enhance the efficacy of adoptively transferred CD8^+^ effector T cells.^[^ ^63^, ^64^ ^]^ PPARγ‐induced FAO boosts the proliferation of CD8^+^ T cells and increases the number of functional CD8^+^ T effector cells, while an upregulation of glycolysis in the T cells by the PPAR agonist has also been observed.^[^ ^63^ ^]^ Under a hypoxic tumor microenvironment, enhanced FAO by PPARα is crucial for the antitumor capacity of CD8^+^ T cells.^[^ ^64^ ^]^ Here, our findings that C24:4 (n‐6)‐PPARα mediates CD8^+^ T cell dysfunction by promoting FAO while reducing glycolysis are from CD8^+^ T cells infiltrated in lung metastatic tissues of breast tumors. These seemingly contradictory studies may reflect the differences in T cell metabolism and function in different tumour types and/or metastasis sites. Under these circumstances, further comprehensive understanding of the molecular mechanisms that regulate the metabolic reprogramming and differentiation fate of T cells appears to be of great significance. Precisely targeting molecular events underlying these mechanisms could potentially restore the immune ability of CD8^+^ T cells and lead to improvements in T cell‐based therapies.

Patients with lung metastases are significantly more likely to have a poor prognosis and diminished therapeutic responsiveness. Treatment for lung metastases remains a therapeutic challenge and is not at all satisfactory. Our findings establish the potential of PLA2G16 as a therapeutic target and a prognostic marker that works well for lung metastasis of breast cancer, with potential applications to other types of tumors. In addition, targeting PLA2G16 and combining with anti‐PD‐1‐based immunotherapy effectively restores the immune response of CD8^+^ T cells and decreases breast cancer lung metastasis in mouse models. Overall, our work may provide an appealing therapeutic strategy against BC lung metastasis.

Finally, the field's understanding of T cell exhaustion has evolved to reveal it as a distinct lineage, comprising multiple subsets with divergent self‐renewal capacity, effector potential, and therapeutic responsiveness, as defined by sophisticated transcriptional and epigenetic landscapes. The precise positioning of the lipid‐induced T cells within this established exhaustion hierarchy remains to be fully elucidated. Future studies employing single‐cell RNA‐sequencing will be crucial to map the developmental relationships and the comprehensive transcriptional network governed by factors in this specific context, thereby providing a resolution that matches the complexity of the exhaustion process.

## 4 Experimental Section

### Cell Culture

The human Triple‐negative breast cancer (TNBC) cell lines and mouse TNBC cell lines were purchased from the American Type Culture Collection (ATCC, USA). MDA‐MB‐231, HCC1806 BT‐549 and 4T1 cells were maintained in RPMI 1640 medium (Gibco, USA), supplemented with 10% fetal bovine serum (Gibco, USA) and 1% streptomycin/penicillin (Beyotime, Shanghai, China). EMT6, E0771 and HS‐578T cells were grown in DMEM medium (Gibco, USA), supplemented with 10% fetal bovine serum (Gibco, USA) and 1% streptomycin/penicillin (Beyotime, Shanghai, China). PY8119 cells were cultured in F‐12K medium (Gibco, USA) with 5% fetal bovine serum (Gibco, USA). All cells were cultured in a 37 °C incubator with 5% CO_2_.

### Stable Cell Line Construction

To construct cell lines with stable knockdown of human *PLA2G16* and murine *Pla2g16*, synthesized shRNA against the target gene and control shRNA were inserted into the pGLV3/H1/GFP/Puro vector or the U6/Luciferase17/Puro vector by GenePharma (Shanghai, China). The detailed shRNA sequences used in this study are listed in Table S1 (Supporting Information). To establish overexpression cell lines, human *PLA2G16* and murine *Pla2g16* sequences were synthesized and cloned into LV17/EF‐1aF/Luciferase17/Puro vector by GenePharma (Shanghai, China). The lentiviruses were infected into TNBC cells following the manufacturer's instructions. The infected cells were further treated with 2 µg mL^−1^ puromycin (Gibco, USA) for 2 weeks to obtain the stably engineered cells.

### Clinical Tissue Specimens and Blood Samples

The primary tumor tissues, paired normal tissues (at least 5 cm distant), and lung metastasis tissues were obtained from breast cancer patients at the First Affiliated Hospital of Chongqing Medical University. The clinicopathologic information of patients was acquired from medical records in accordance with institutionally approved procedures. Samples of whole blood for CD8^+^ T cell isolation were collected from healthy donors at the First Affiliated Hospital of Chongqing Medical University. This study was approved by the Ethics Committee of Chongqing Medical University.

### Animal Models

BALB/c and C57BL/6J mice (female, 6‐8 weeks old) were purchased from Chongqing Medical University Laboratory Animal Center (Chongqing, China), and BALB/c‐nude mice (female, 6‐8 weeks old) were purchased from Gempharmatech (Sichuan, China). Pparα^−/−^ C57BL/6J mice (8‐week‐old, female) were purchased from Cyagen Bioscience (Suzhou, China). Animal studies were approved by the animal care ethics committees of Chongqing Medical University and conducted in accordance with the guidelines for the care and use of laboratory animals.

To establish the orthotopic breast cancer model, 1×10^6^ EMT6 (shCtrl/shPLA2G16) cells, PY8119 (PY8119/PLA2G16) cells, and MDA‐MB‐231 (shCtrl/shPLA2G16) in 100µL PBS were inoculated into the fourth mammary fat pads of 6‐week‐old female BALB/c mice, C57BL/6J mice, and BALB/c‐nude mice, respectively. Tumor sizes were determined every 3 days by caliper measurements, and tumor volume was calculated by the equation: volume = (length × width^2^)/2. At 21 days after tumor inoculation, the primary tumors were surgically removed to allow for better observation of distant metastasis. For the intravenous metastatic model, 3 × 10^5^ EMT6 (shCtrl/shPLA2G16) cells or PY8119 (LEV/PLA2G16) cells in 30µL PBS were injected into the lateral tail vein of BALB/c, or in C57BL/6 mice, respectively. Mouse health and weight were monitored every other day. For LEI110 treatment, C57BL/6J mice were randomized and intraperitoneally administered with LEI110 (5 mg kg^−1^, once every two days). For GW6471 or etomoxir (ETO) treatment, 10 mg kg^−1^ of GW6471 or 30 mg kg^−1^ of ETO was administered intraperitoneally once every two days until the mice were sacrificed. For mouse PD‐1 blockade antibody treatment, BALB/c mice were given an intraperitoneal injection of 10 mg kg^−1^ of mouse PD‐1 blockade antibody every 2 days. For CD8^+^ T cells depletion, BALB/c mice received treatment intraperitoneally with 200 µg/mouse of anti‐mouse CD8α (BioXCell, USA) or rat IgG (BioXCell, USA) once every 3 days before intravenous injection of TNBC cells, and then the treatment was maintained with 100 µg/mouse twice a week after cancer cell injection. To perform bioluminescent imaging (BLI), mice were anesthetized and injected intraperitoneally with 150 mg kg^−1^ of substrate D‐luciferin (Sciencelight, China). The Small Animal Living Imaging System (Berthold LB983, Germany) was used to capture imaging pictures and living image software version 2.50 was applied to calculate the photon flux.

### Mouse CD8^+^ T Cells Isolation and Activation

Spleens harvested from mice were digested into a single cell suspension, and the red blood cells were lysed with RBC lysis buffer (Biosharp, China). CD8^+^ T cells were then sorted by positive selection kit (CD8a (Ly‐2) MicroBeads) following the manufacturer's instructions (Miltenyi Biotec, Germany). CD8^+^ T cells were then resuspended in complete RPMI 1640 medium (10% FBS, 1% NaPy, 1% Glutamine, 0.1% β−mercaptoethanol) at a density of 1 × 10^6^ cells mL^−1^. While for activation, CD8^+^ T cells were activated in the medium with 5 µg mL^−1^ plate‐bound αCD3 (MULTI SCIENCES, China) and 5 µg mL^−1^ soluble αCD28 (MULTI SCIENCES, China) for 48 h in a 37 °C incubator with 5% CO2. For some experiments, cells were further treated with PBS, free fatty acid (FFA) C24:4 (n‐6), FFA C20:4, FFA C22:4 or co‐cultured with TNBC engineered cells. Unless otherwise mentioned, the ultimate concentration for each FFA was maintained at 100 µm, and the number of TNBC cells was 5 × 10^5^. Unless other‐wise specified, cells were activated for 48 h at 37 °C, 5% CO_2_.

### Flow Cytometry

For cell surface staining, tumor tissues, lung tissues or fresh spleens were harvested, and the single‐cell suspension was obtained as described before.^[^ ^29^ ^]^ Cells were first incubated in stain buffer (BD Pharmingen, USA) with Fc block antibody at 4 °C for 20 min and then stained with fluorescently labeled antibodies at the recommended dilution at 4 °C for 30 min in the dark. Then cells were washed twice with DPBS and resuspended in stain buffer.

For measurement of intracellular cytokines production in mouse or human primary CD8^+^ T cells, cells were first stimulated with Cell Stimulation Cocktail (eBioscience, USA) at 37 °C for 5 h. Cells were harvested, washed twice with DPBS, and conducted with surface staining. Then, cells were fixed, permeabilized with Intracellular Fixation/Permeabilization Buffer Kit (Elabscience, China) or Transcription Factor Staining Buffer Set (eBioscience, USA), and stained with antibodies against indicated cytokines at 4 °C for 30 min in the dark. Stained cells were washed 2 times with DPBS and resuspended in staining buffer prior to FACS analysis. Cells were first gated using FSC/SSC characteristics to exclude debris, and then target cell population of interest was gated by specific stain. Data were analyzed by using FlowJo v10.0 (FlowJo, LLC, Ashland, OR, USA). The following antibodies were used: anti‐mouse CD16/CD32 (#553141, BD Pharmingen), APC Cy7 anti‐mouse CD45 (#557659, BD Pharmingen), PerCP/Cy5.5 anti‐mouse CD3e (#561108, BD Pharmingen), BV510 anti‐mouse CD4 (#563106, BD Pharmingen), FITC anti‐mouse CD8a (#561966, BD Pharmingen), APC anti‐mouse PD‐1 (#562671, BD Pharmingen), PE/Cy7 anti‐mouse CD44 (#560569, BD Pharmingen), APC anti‐human/mouse Granzyme B (#396408, Biolegend), PE/Cy7 anti‐mouse TNF‐α (#506324, Biolegend), PE anti‐mouse TIM3 (#134003, Biolegend), PE anti‐mouse IFN‐γ (#505808, Biolegend), PE anti‐human/mouse Ki‐67 (#151210, Biolegend), APC/Cy7 anti‐human CD8 (#557834, BD Pharmingen), FITC anti‐human CD3 (#555339, BD Pharmingen), PEanti‐human IFN‐γ (#502509, Biolegend), BV650 anti‐human TNF‐α (#563418, BD Pharmingen), APC anti‐human TIM3 (#345012, Biolegend), PE anti‐human PD‐1 (#567617, BD Pharmingen).

### RNA Preparation and qRT‑PCR

Total RNA was isolated from tumor tissues, TNBC cells or mouse CD8^+^ T cells by TRIzol reagent (Takara, Japan) and reverse transcribed into cDNA by the PrimeScript RT Reagent Kit (Takara, Japan). The SYBR Premix Ex Taq II kit (Takara, Japan) was utilized for quantitative real‐time PCR (qRT‑PCR) to detect indicated gene expression. The primers used in this study are listed in Table S1 (Supporting Information).

### Lactate Assay

CD8^+^ T cells were treated with or without C24:4 (n‐6) for 48 h. The cultural medium supernatant was collected and centrifuged at 2000 rpm for 15 min to remove any cell debris. Then, the Lactate Colorimetric/Fluorometric Assay Kit (Abcam, UK) was used to determine the lactate concentration in the medium according to the manufacturer's instructions.

### Western Blotting Analysis

TNBC cells and tumor tissues were harvested, washed, and lysed in RIPA buffer (Beyotime, China) to extract total protein. Total protein was quantified with BCA protein assay kit (Beyotime, China). Appropriate amounts of proteins (30–50 µg/well) were separated by 8%–12% SDS‐PAGE gel and transferred to PVDF membranes (Bio‐Rad, USA). The membranes were blocked in 5% defatted milk (BOSTER, China) at room temperature for 1 h and incubated with the specific primary antibodies at 4 °C overnight. The following primary antibodies were used in this study: PLA2G16 (1:500, A16018, ABclonal), PLA2G16 (1:1000, #ABIN2840461, Antibodies online), PPARα (1:1000, ab126285, Abcam), β‐Actin (1:1000, BL071A, Biosharp). The membranes were then incubated with the appropriate horseradish peroxidase (HRP)‐conjugated secondary antibodies for 2 h at room temperature, and the enhanced chemiluminescence system (Bio‐Rad, Hercules, CA, USA) was used to detect the protein bands. Images were captured by Scion image software and β‐Actin was utilized as an internal control.

### Cell Viability Assay

Cell viability was detected by the Cell Counting Kit‐8 (Beyotime, China). Briefly, TNBC cells (1500 cells per well) were seeded into 96‐well plates and cultured for 24 h, 48 h or 72 h. CCK‐8 solution (10 µL /well) was added to the plate at indicated time point and incubated for 3 h, and absorbance values were measured at 450 nm by a microplate reader (BioTek, Winooski, VT, USA).

### Hematoxylin & Eosin (H&E) and Immunofluorescence (IF) Staining

For histopathology, tumor tissues and lung metastasis tissues from TNBC patients or mice were collected at the designed days, dipped in 4% formalin‐fixed overnight, paraffin embedded, and 4–5 µm‐thick sections were obtained by microtome, and then stained with haematoxylin and eosin (H&E). H&E‐stained tissue samples were measured using a Zeiss Axio Imager M2m microscope. Images were captured by a 10x Plan‐Neofluar NA 0.3 objective, then processed with software. For immunofluorescence, the slides were incubated in 3% H_2_O_2_ solution, blocked with 10% goat serum, and incubated with primary antibodies at 4 °C overnight in the dark. Information of all antibodies used in this study is as follows: PLA2G16 (1:100, #ABIN524566, Antibodies online), CD8α (1:100, #ab237709, Abcam), cleaved caspase3 (1:100, #9661, Cell Signaling Technology). Then, the slides were washed with PBS for 3 times, incubated with immunofluorescence fluorescent secondary antibody at room temperature for 30 min, and mounted by mounting medium with DAPI. Images were captured by OLYMPUS cellSens standard software (version 1.11) and analyzed by Caseviewer2.3 software.

### Immunohistochemical (IHC) Staining

Paraffin‐embedded sections were de‐waxed, rehydrated, and blocked for endogenous peroxidase and nonspecific binding sites. The sections were then incubated with rabbit anti‐PLA2G16 (1:100, Sangon Biotech) polyclonal antibody overnight at 4 °C. Subsequently, the slides were incubated with polyperoxidase‐anti‐rabbit IgG (ZSGB‐BIO, China) for 30 min at 37 °C and then stained with diaminobenzidine. After counterstaining by hematoxylin and sealed, images were captured using a Nikon Eclipse 80i microscope (Tokyo, Japan).

### Electron Microscopy

CD8^+^ T cells were first prefixed with 3% glutaraldehyde. Then CD8^+^ T cells were post‐fixed in 1% osmium tetroxide, dehydrated in a series acetone, infiltrated in Epon 812, and embedded. The semithin sections were stained with methylene blue, and ultrathin sections were cut with a diamond knife, stained with uranyl acetate and lead citrate. Sections were analyzed, and imaging pictures were taken by JEM‐1400‐FLASH Transmission Electron Microscope (JEOL, Japan).

### Isolation of Primary Human CD8^+^ T Cells

Whole blood from healthy donors was used for peripheral blood mononuclear cells (PBMC) isolation by Ficoll density separation. CD8^+^ T cells were isolated from PBMCs by CD8^+^ T cell isolation kit (Miltenyi Biotec, Germany) and were further cultured in complete RPMI 1640 medium supplemented with 10% FBS and 100 U mL^−1^ IL‐2. For CD8^+^ T cells activation, CD8^+^ T cells were activated with immobilized anti‐human CD3 antibodies (2 mg mL^−1^, #551916, BD Pharmingen) and anti‐human CD28 antibodies (2 mg mL^−1^, #556620, BD Pharmingen) and cultured for 48 h in the presence or absence of FFA C24:4 (n‐6). Cytokines (IFN‐γ, TNF‐α, and granzyme B) production and PD‐1 and TIM3 expression were further determined by FACS analysis.

### RNA‐Seq and Bioinformatics Analysis

For RNA Sequencing, RNA was first extracted from purified control CD8^+^ T cells and C24:4 (n‐6)‐treated CD8^+^ T cells with TRIzol reagent (Takara, Japan). The quality and quantity of the total RNA were verified with a Nano Drop and Agilent 2100 bioanalyzer (Thermo Fisher Scientifc, MA, USA), and RNA library construction and subsequent RNA sequencing were performed by BGI (Shenzhen, China). The sequencing data were filtered with SOAPnuke (v1.5.2) and the clean reads were mapped to the reference genome using HISAT2 (v2.0.4). After aligning the clean reads to the reference coding gene set using Bowtie2 (v2.2.5), RSEM (v1.2.12) was applied to calculate the gene expression levels. Differential expression analysis was essentially carried out with the DESeq2 (v1.4.5) with q‐value (false discovery rate (FDR) adjusted *P*‐value ≤ 0.05. GO (http://www.geneontology.org/) and KEGG (https://www.kegg.jp/) enrichment analysis of annotated differentially expressed genes was performed by Phyper (https://en.wikipedia.org/wiki/Hypergeometric_distribution) based on hypergeometric test. All above analyses were performed on the Dr. Tom analysis system constructed by BGI (Shenzhen, China). GSEA (https://www.broadinstitute.org/gsea/index.jsp) was used for further analysis of the identified gene set involved in a specific metabolic pathway.

### Lipid Extraction

For tumor cells culture medium, the same number of MDA‐MB‐231 (MDA‐MB‐231/shCtrl and MDA‐MB‐231/shPLA2G16) cells were cultured in culture medium without lipid addition. 2 mL culture fluid was collected and centrifuged at 400 g, 4 °C for 10 min to remove cell debris. For the collection of lung interstitial fluid, BALB/c female mice with EMT6 (shCtrl/shPLA2G16‐2) cells via intravenous injection were first euthanized, and subsequently, lungs were harvested, washed with pre‐cooled PBS, and dried by carefully tapping on a gauze. Then, the lungs were placed in a homemade filtered centrifuge tube with a nylon mesh filter with 20 µm opening pores (Repligen, USA), and the lung interstitial fluid was collected by centrifugation at 400 × g, 4 °C for 10 min. All the samples were snap frozen in liquid nitrogen, and stored at −80 °C.

### Metabolomics and Lipidomic Assays

Liquid chromatography‐MS lipidomic analyses were performed by ultra high‐performance liquid chromatography tandem quadrupole time of flight mass spectrometry (UHPLC‐QTOF‐MS). Waters ACQUITY UPLC BEH Amide (2.1 mm × 100 mm, 1.8 µm) was applied for HPLC separation, and an ACQUITY Premier series UHPLC system was used to perform the UHPLC separation. Assay development was performed using a SCIEX Triple Quad 6500+ mass spectrometer, and the quantification of the target compounds was conducted by Biobud‐v2.1.4.1 software. The absolute content of individual lipids corresponding to the IS was calculated on the basis of peaks area and the actual concentration of the identical lipid class internal standard (IS).

### Cellular Energy Metabolism Analysis

Energy metabolic profile of CD8^+^ T cells was measured by the Seahorse XF‐96e extracellular flux analyzer (Seahorse Bioscience, Agilent). Briefly, CD8^+^ T cells (3 × 10^5^ /per well) were treated with or without C24:4 (n‐6) for 48 h before XF analysis and mitochondrial perturbation experiments were conducted by the MitoStress Test Kit (Agilent, USA) according to the manufacturer's instructions. Oxygen consumption rates (OCR, pmol min^−1^) and extracellular acidification rates (ECAR, mpH min^−1^) were recorded in real time following the sequential treatment with oligomycin (Oligo, 1 µM), FCCP (1.5 µM), and Rot/AA (Rotenone + antimycin A, 0.5 µM). An adaptation of the standard MitoStress Test methodology was employed to evaluate the contribution of fatty acid oxidation (FAO) to CD8^+^ T cell OCR levels. Briefly, 200 mM of etomoxir (Sigma, Germany) acute injection was performed ahead of the regular MitoStress Test. OCR, ECAR levels were recorded, and FAO levels were calculated following the manufacturer's protocol.

### Statistical Analysis

The data presentation and statistical analyses are described in the figure legends. Statistical analysis was performed with GraphPad Prism 8.0 software (San Diego, CA, USA). *p* < 0.05 was assumed as statistically significant. The experiments in this study were set up using 3–12 samples or animals per independent group, condition or biological replications, and statistics are indicated in the figure legends.

### Ethics Approval Statement and Patient Consent Statement

All human tissue samples were collected in accordance with national and institutional ethical guidelines. The use of human samples in this study was reviewed and approved by the Ethics Committee of Chongqing Medical University (Reference number: 2023048). All participants provided written informed consent before their inclusion in the study. All animal procedures performed in the project were approved by the Institutional Animal Care and Use Committee of Chongqing Medical University (Approval Number: IACUC‐CQMU‐2023‐0198).

## Conflict of Interest

The authors declare no conflict of interest.

## Author Contributions

Y.G., D.M., L.L. and J.L. contributed equally to this work. M.L., Y.G. and D.T. designed this study; Y.G., D.M., L.L. and J.L. performed most of the experiments. P.D., Z.L., Y.G., R.W., D.T., Y.H and X.T. carried out the animal experiments. P.D., C.C., Y.H and S.C. analyzed the data. Y.G., J.L., C.C. and S.C. collected patient samples. Y.G, D.M., L.L. and J.L. organized the figures and drafted the initial manuscript; M.L. and D.T. revised this manuscript. All authors read and approved the final manuscript.

## Supporting information



Supporting Information

Supporting Information

Supporting Information

## Data Availability

The data that support the findings will be available in NCBI Sequence Read Archive (SRA) at https://dataview.ncbi.nlm.nih.gov/object/PRJNA1353566?reviewer=9gf03pht68v29fre8eoakksji1 following an embargo from the date of publication to allow for commercialization of research findings.
