# Supplementary material for: PLA2G16‐Mediated Tetracosatetraenoic Acid Rewires Fatty Acid Oxidation to Impair CD8+ T Cell Immune Function in Promoting Breast Cancer Lung Metastasis
Source: Adv Sci (Weinh). 2025 Nov 16;13(6):e10224. doi: 10.1002/advs.202510224 (PMC12866795; doi:10.1002/advs.202510224)
Supplement: Supplementary file 1 — Supporting Information [file ADVS-13-e10224-s002.docx]

**Supplementary Figure legends**

**
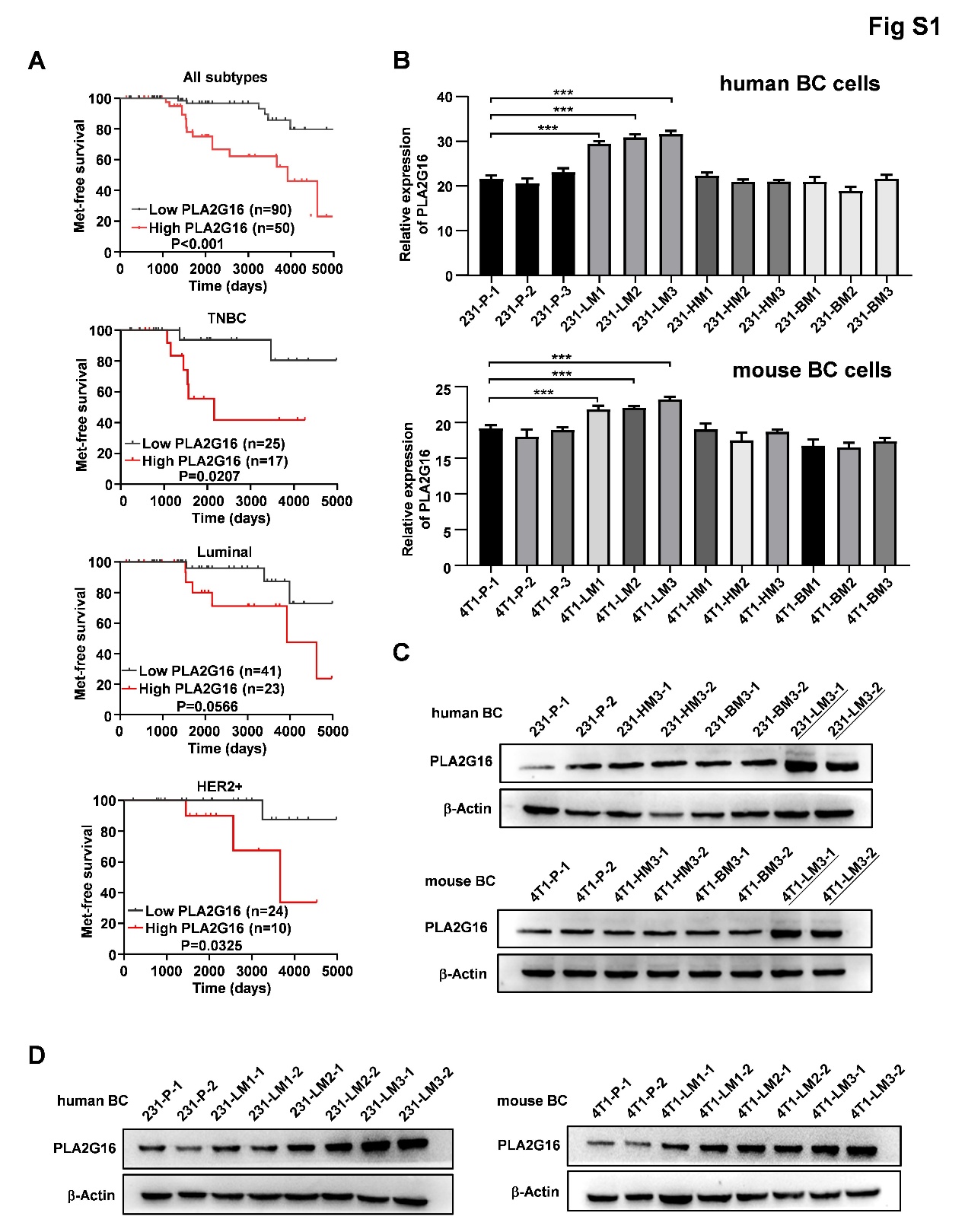
**

**Supplementary Figure 1. PLA2G16 is required for breast cancer lung metastasis (Related to Figure 1)**

**A.** Kaplan-Meier metastasis-free survival plot of breast cancer patients. All subtypes, TNBC, Luminal and HER2^+^ based on PLA2G16 expression in cohort. **B.** Relative folds of Pla2g16 mRNA expression in metastatic MDA-MB-231 or 4T1 derived from various organ lesions and parental cells were detected by qRT-PCR (P: parental; LM1/HM1/BM1: the first lung/liver/brain metastatic cells; LM2/HM2/BM2: the second lung/liver/brain metastatic cells; LM3/HM3/BM3: the third lung/liver/brain metastatic cells.) (n = 3). **C.** Western blot analysis of PLA2G16 protein levels in metastatic MDA-MB-231 or 4T1 derived from various organ lesions and parental cells. **D****.** Western blot analyses of PLA2G16 expression in the indicated parental and derivative cells (LM1/LM2/LM3). Data are the mean ± SD. *p < 0.05, **p < 0.01, and ***p < 0.001 by log rank test in (A) or two-sided unpaired Student’s t test in (B). n.s., not significant.

**
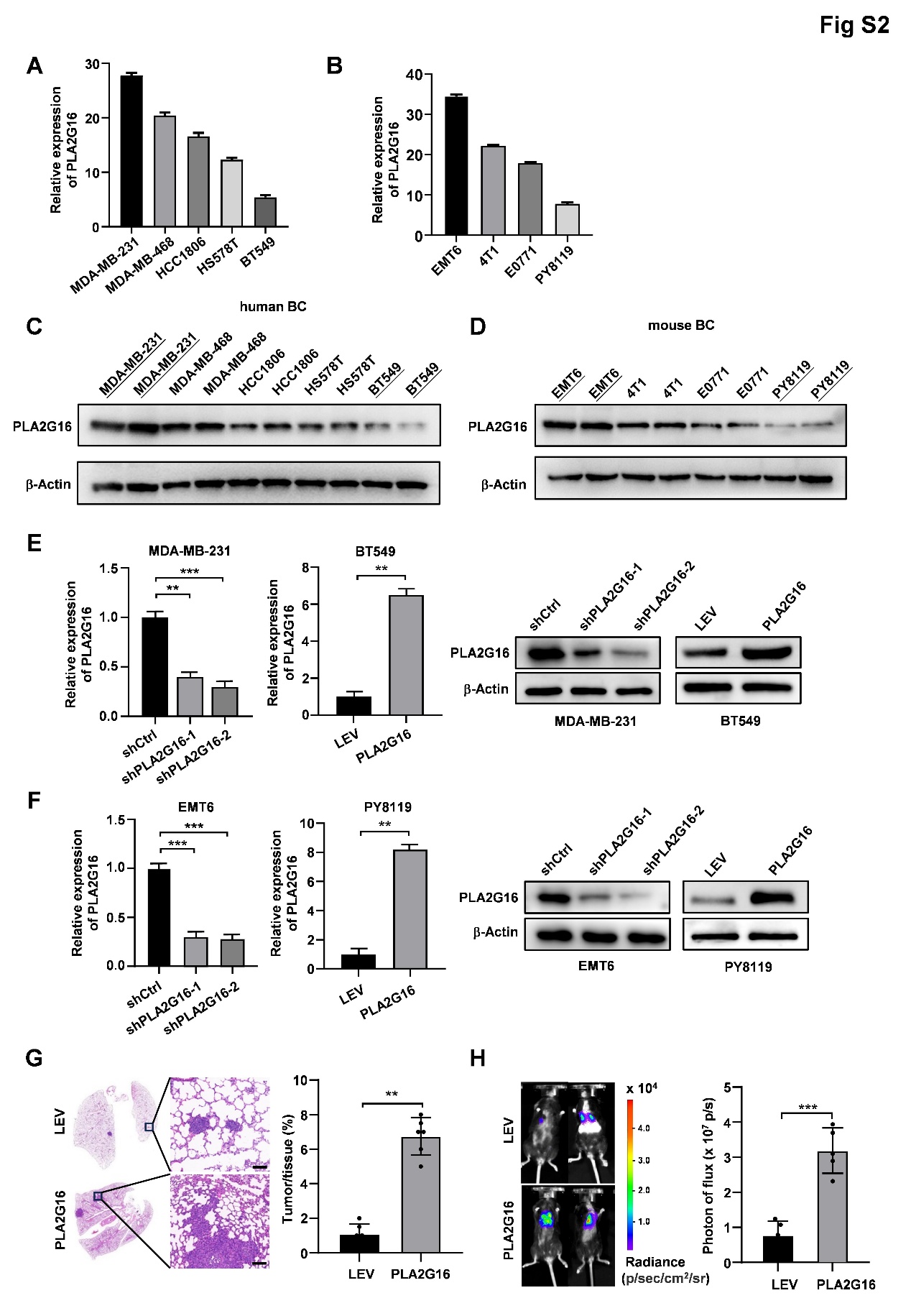
Supplementary Figure 2. Construct cell models with PLA2G16 stable knockdown or overexpression**

**A-D.** qRT-PCR (A-B) and western blotting (C-D) analysis of PLA2G16 expression in representative human triple-negative breast cancer cells (n = 3) and mouse breast cancer cells (n = 3). **E-F.** qRT-PCR (n = 3) and western blotting analysis of PLA2G16 expression in human (C) and mouse (D) breast cancer cells transfected with shCtrl, shPLA2G16-1, and shPLA2G16-2 or the indicated tumor cells transfected with control lentivirus (LEV) and PLA2G16-overexpressed lentivirus (PLA2G16). **G.** Representative H&E-stained sections of lung metastasis (left) and metastatic burden (right) in C57BL/6J mice (n = 6) (Scale bars, 100 μm). **H.** Intravenous injection of PY8119 with or without ectopic PLA2G16 for lung colonization analysis. Shown are the representative images of BLI and quantification of metastases (n = 5). Data are the mean ± SD. *p < 0.05, **p < 0.01, and ***p < 0.001 by two-sided unpaired Student’s t test. n.s., not significant.

**
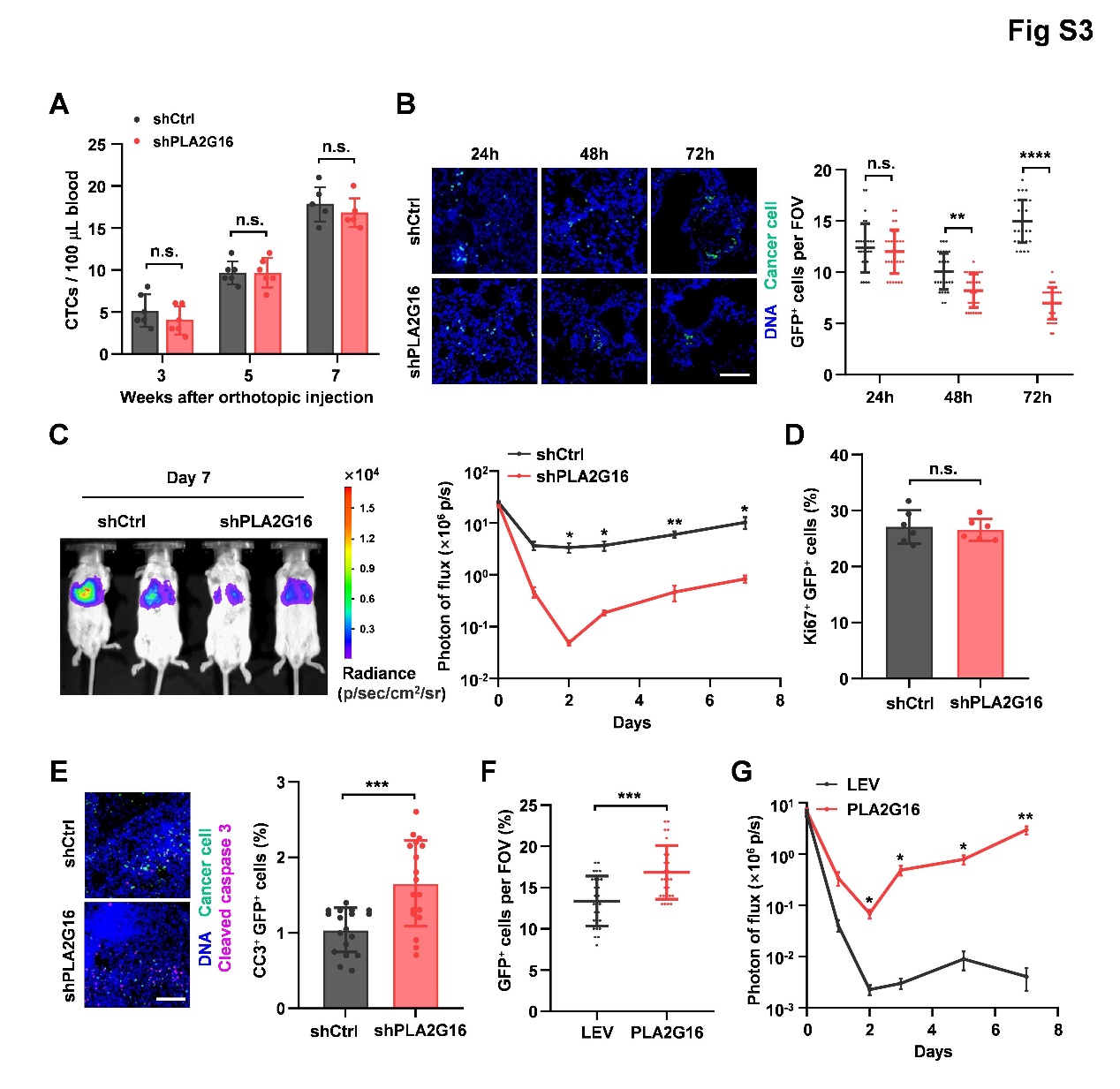
Supplementary Figure 3.** **PLA2G16 promotes DTC survival in early stage and sustains outgrowth of breast cancer cells**

**A.** Frequencies of circulating tumor cells (CTCs) in the peripheral blood of PLA2G16 wild type or knockdown EMT6 tumor-bearing mice at 3-, 5- or 7-weeks post injection (n = 6 mice per group for week 3 and week 5; n = 5 mice per group for week 7). **B-C.** Seeding number of PLA2G16 knockdown or control EMT6 cells in lung and formation of lung metastasis at the early stage after intravenous injection. Shown are IF analyses of GFP^+^ cancer cells in lung sections (B, n = 30 RMFs; FOV: field of view under microscope) from 3 mice of each group) (Scale bars, 50 μm) and BLI quantification within week 1 (C, n = 4). **D.** The percentage of Ki67^+^GFP^+^ DTC cells in mice lung at 72 h after injection was assessed by FACS analysis (n = 6). **E.** Representative images and quantification of the cleaved caspase 3-positive DTC cells in lungs at 72 h after injection (n = 18 RMFs from 3 mice of each group) (Scale bars, 50 μm). **F-G.** Seeding number of PLA2G16 over-expressed or control PY8119 cells and formation of lung metastasis at the early stage after intravenous injection. Shown are IF analyses of total cancer cells seeded in lungs at 72 h (F, n = 27 RMFs from 3 mice of each group) and BLI analyses in week 1 (G, n = 4). Data are the mean ± SD. *p < 0.05, **p < 0.01, and ***p < 0.001 by two-tailed unpaired t test (D), (E) and (F) or two-way ANOVA in (others). n.s., not significant.

**
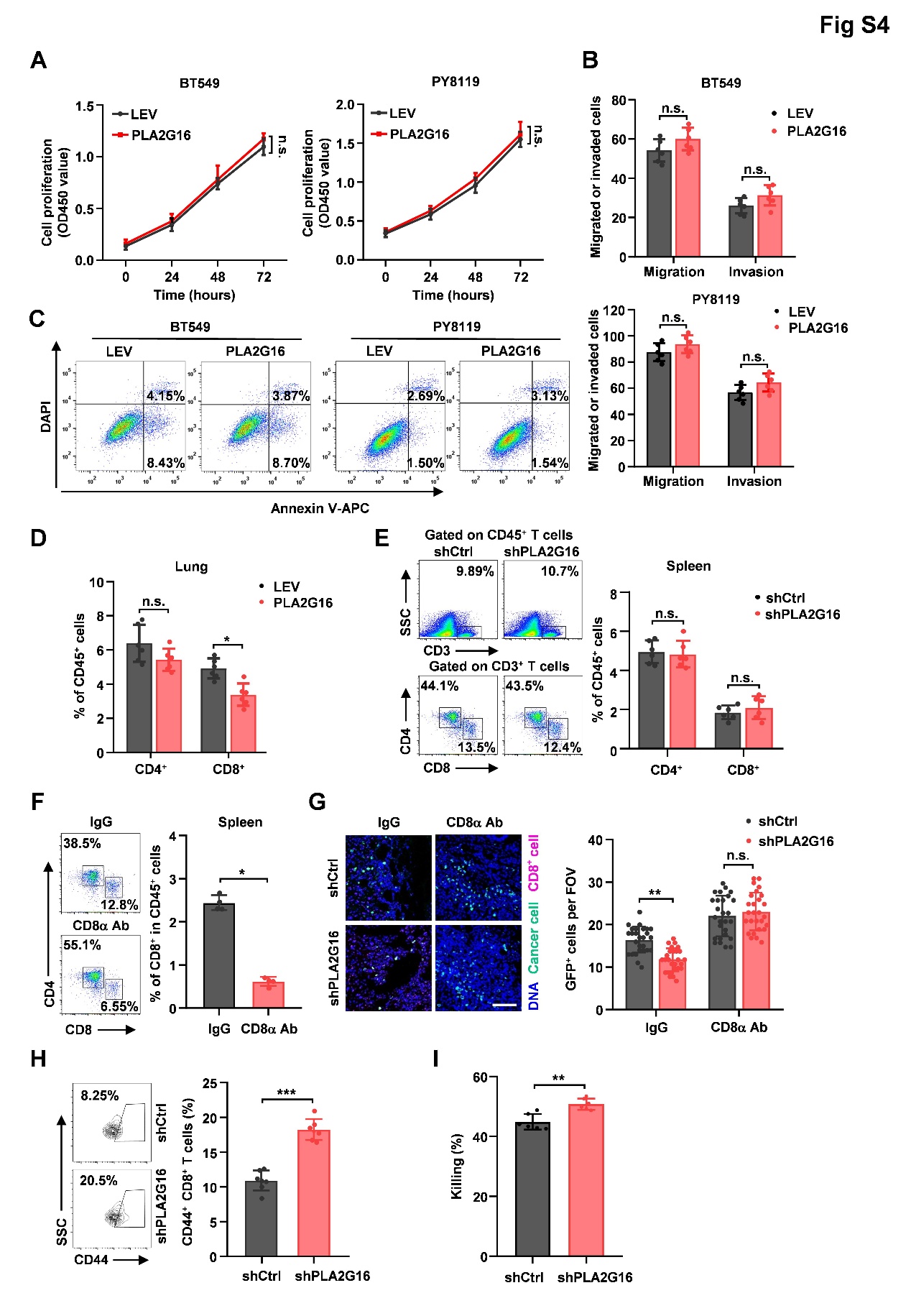
Supplementary Figure 4. Tumoral PLA2G16 inhibits CD8^+^ T cell activation and clonal expansion in lung metastasis niches (Related to Figure 3)**

**A-C.** In vitro analyses of tumor cell proliferation (A), migration or invasion (B) and apoptosis (C) of PLA2G16 overexpressing and control tumor cells (n = 3 in (A), or n = 6 in (B) from biologically independent samples). **D.** The ratio of CD8^+^ T cells and CD4^+^ T cells in CD45^+^ immunocytes from lung parenchyma (n=6 mice per group) was determined by FACS analysis. **E.** Flow cytometry analyses of CD8^+^ T cells in CD45^+^ immunocytes in spleen at 72 h after mice injected with PLA2G16 knockdown or parental EMT6 cells (n = 6 mice per group). **F.** Flow cytometry analyses of CD8^+^ T cells in control or anti-CD8-treated mouse spleen (n = 4 mice per group). **G.** IF analyses of tumor cells in lungs of mice treated with IgG or the CD8^+^ T cell clearance antibody at 72 h after intravenous EMT6 injection (n = 27 RMFs from 3 mice per group) (Scale bars, 50 μm). **H.** Balb/c mice were intravenously injected with PLA2G16 knockdown or control EMT6 cells. The percentage of CD44^+^ CD8^+^ T cell in the lung was assessed (n = 7 in H, n = 8 in I or n = 9 in J). **I.** Killing efficiency of CD8^+^ T cells isolated from mice injected with PLA2G16 knockdown or parent EMT6 cells. Analysis was performed after 24 hours (n= 6). Data are the mean ± SD. *p < 0.05, **p < 0.01, and ***p < 0.001 by two-tailed unpaired t test. n.s., not significant.

**
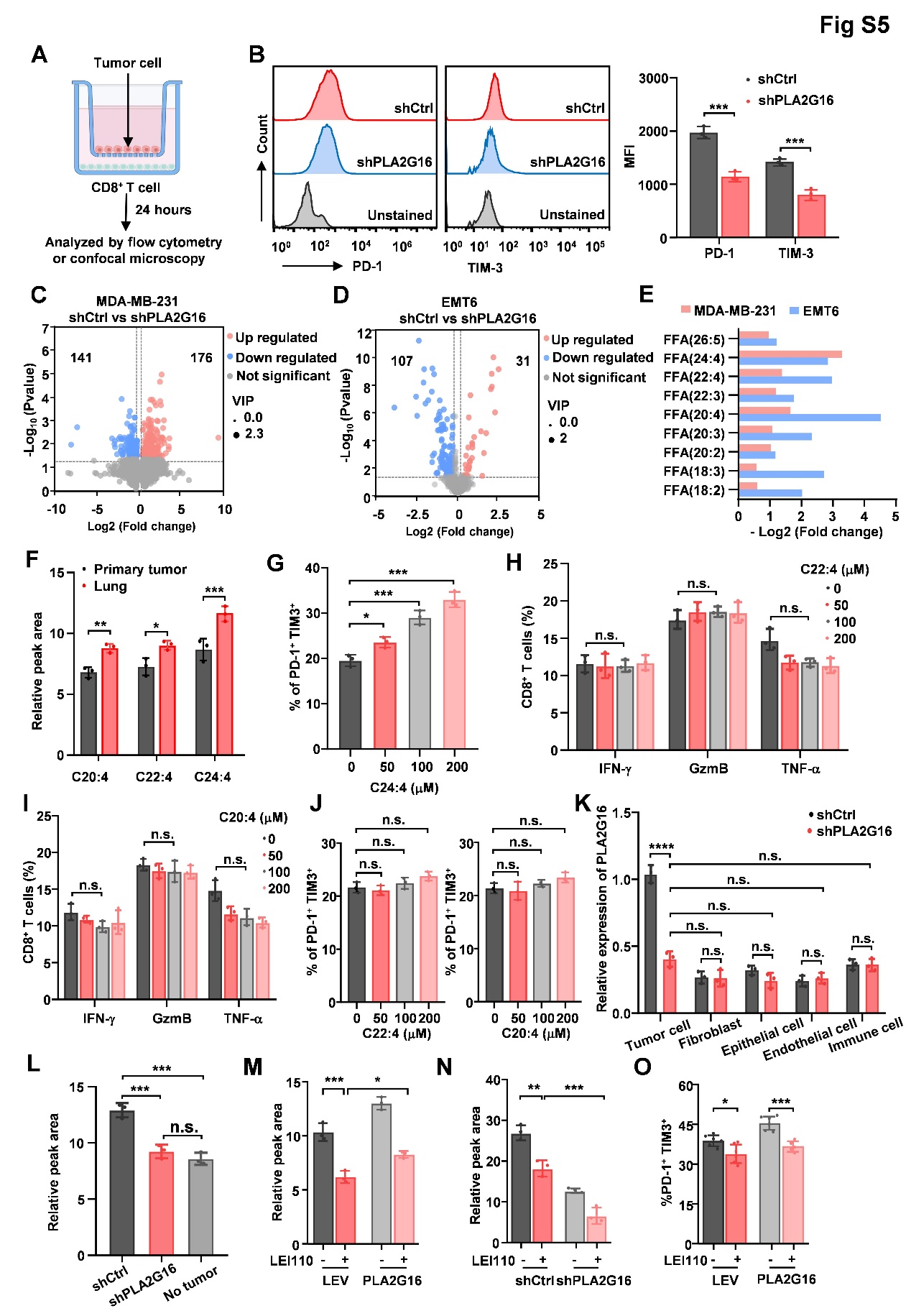
Supplementary Figure 5. PLA2G16 expression in cancer cells mediates the accumulation of C24:4 (n-6) (Related to Figure 3)**

**A.** Experiment scheme for co-culture of CD8^+^ T cells with EMT6 engineered cells. **B.** Fluorescence-activated cell sorting (FACS) analysis of PD-1 and TIM3 expression in CD8^+^ T cells co-cultured with EMT6 engineered cells (n = 3). **C-D.** Volcano plot representing changes of detected compounds in shCtrl versus shPLA2G16 in MDA-MB-231 cell-conditioned medium (C) and lung interstitial fluid (D) from EMT6 metastatic mice. Number of metabolites that met significant criteria (P < 0.05, fold change ≥ |1.5|) is indicated. **E.** The top 9 different FFAs detected in MDA-MB-231 cell-conditioned medium and tumor metastatic lung interstitial fluid. **F.** The content of C20:4, C22:4 and C24:4 (n-6) in primary tumor and lung metastases were detected. **G.** Mouse naive CD8^+^ T cells were treated with or without C24:4 (n-6) and exhaustion profile assessed as PD-1 and TIM3 expression levels (n = 3). **H-I.** The activation of mouse naive CD8^+^ T cells treated with or without free fatty acid C22:4 (H) and C20:4 (I) was assessed by cytokines production (n = 3). **J.** Mouse naive CD8^+^ T cells were treated with or without C20:4 or C22:4 and their exhaustion profile was assessed by PD-1 and TIM3 expression levels (n = 3). **K.** qRT-PCR analysis of *Pla2g16* mRNA level in tumor cells, fibroblasts, epithelial cells, endothelial cells and immune cells (n = 3). **L.** The contents of C24:4 (n-6) in the lung interstitial fluid of normal mice or mice with EMT6 (shCtrl/shPLA2G16) lung metastases (n = 3). **M.** PLA2G16 overexpressing or control PY8119 cells were pre-treated with or without LEI110 (200 nM), the contents of lipid metabolite C24:4 (n-6) in cell-conditioned medium were measured (n = 3). **N.** PLA2G16 knockdown or control EMT6 cells were pre-treated with or without LEI110 (200 nM), the contents of C24:4 (n-6) in cell-conditioned medium were detected (n = 3). **O.** Mouse CD8^+^ T cells were co-cultured with PLA2G16 overexpressed or control PY8119 cells under pre-treatment with or without LEI110 (200 nM), exhaustion profiles were assessed by PD1 and TIM3 levels (n = 6). Data are the mean ± SD. *p < 0.05, **p< 0.01, and ***p < 0.001 by two-tailed unpaired t test. n.s., not significant.

**
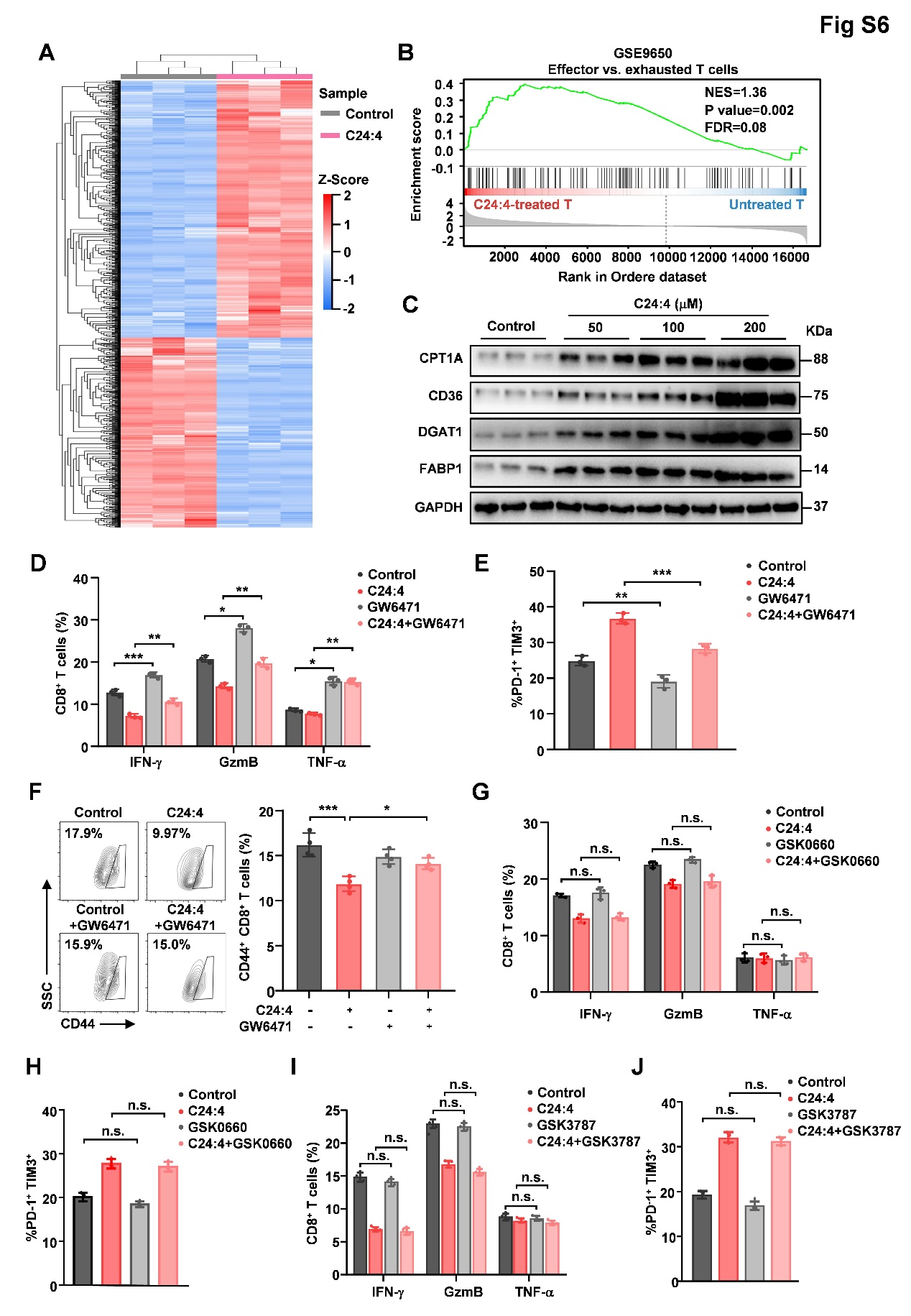
**

**Supplementary Figure 6. PPARα signal is closely associated with CD8^+^ T cell dysfunction and lipid accumulation (Related to Figure 4)**

**A.** Unsupervised clustering analysis of the transcriptome of control and C24:4 (n-6) conditioned mouse naive CD8^+^ T cells (n = 3). Top 1,648 most variable genes were used to plot the heatmap. **B.** GSEA enrichment analysis in C24:4 (n-6)-treated CD8^+^ T cells compared with control CD8^+^ T cells using well-established T cell exhaustion gene sets from published studies (GSE9650). **C.** Mouse naive CD8^+^ T cells were treated with C24:4 (n-6), and the PPARα target proteins, such as CPT1a, DGAT1, CD36 and FABP1, were assessed by western blotting. **D-F.** CD8^+^ T cells were incubated with or without C24:4 (n-6) (100 μM) in the presence or absence of GW6471 (15 μM) for 48 h. The indicated cytokine production (C, n = 3), PD1 and TIM3 levels (D, n = 3) and CD44 expression (E, n = 3) were assessed by FACS analysis. **G-H.** CD8^+^ T cells were incubated with or without C24:4 (n-6) (100 μM) in the presence or absence of GSK0660 (15 μM) for 48 h. The indicated cytokine production (F, n = 3) and PD1 and TIM3 levels (G, n = 3) were assessed by FACS analysis. **I-J.** CD8^+^ T cells were incubated with or without C24:4 (n-6) (100 μM) in the presence or absence of GSK3787 (15 μM) or for 48 h. The indicated cytokine production (H, n = 3) and PD1 and TIM3 levels (I, n = 3) were assessed by FACS analysis. Data are the mean ± SD. *p < 0.05, **p < 0.01, and ***p < 0.001 by two-tailed unpaired t test. n.s., not significant.

**
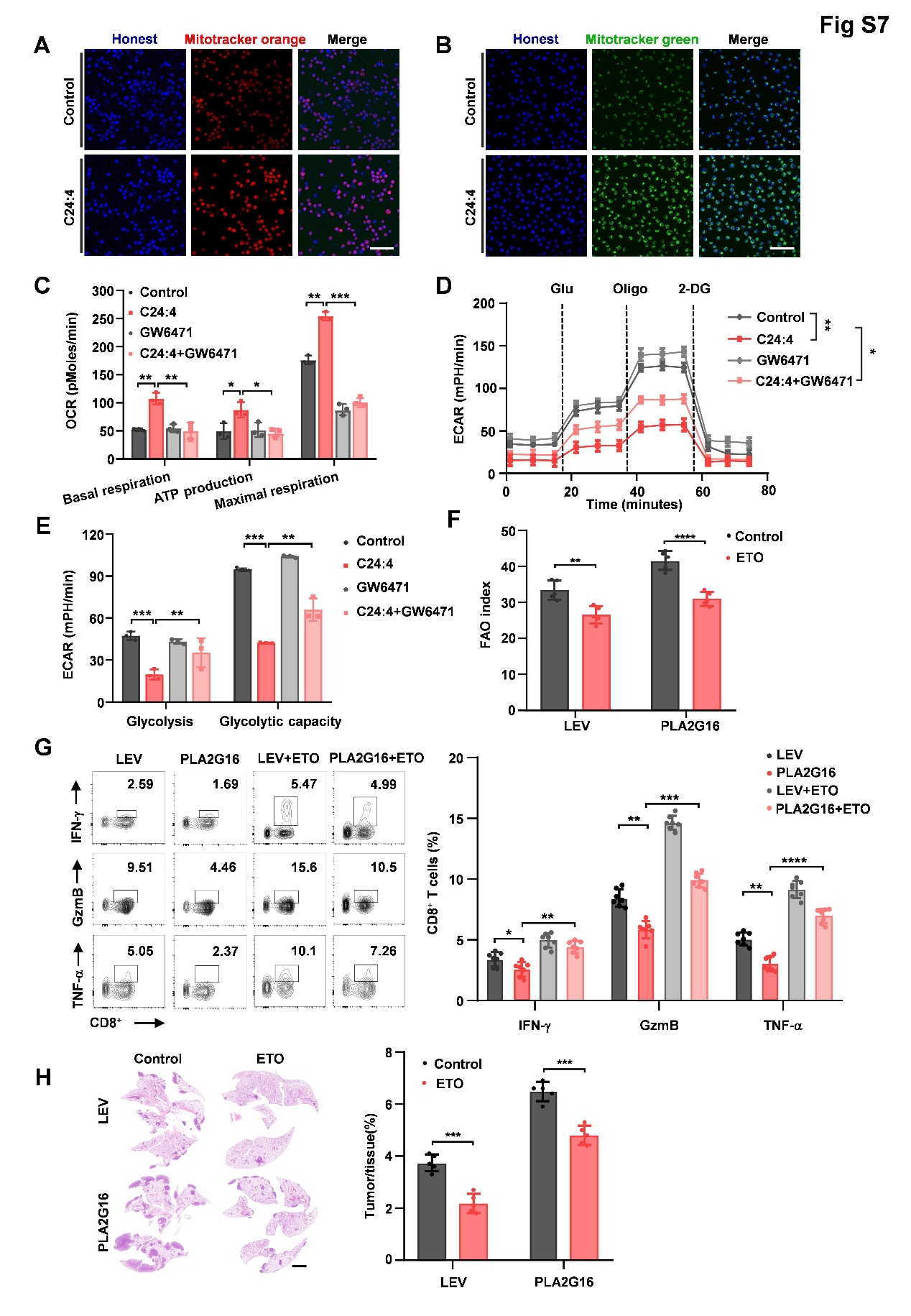
Supplementary Figure 7. C24:4 (n-6)-induced metabolic reprogramming is related to suppression of CD8^+^ T cells**

**A-B.** Mitochondrial membrane potential (A) or Mitochondrial mass (B) based on confocal microscopy analysis of Mitotracker orange (Scale bar, 50 mm) or Mitotracker green (Scale bar, 50 μm). **C.** The basal respiration, ATP production, and maximal respiration were calculated based on the data from Figure 6E (n = 3). **D.** CD8^+^ T cells were pre-treated with or without C24:4 (n-6) (100 mM) in the presence or absence of GW6471 (15 mM) for 48 h and ECAR was recorded (n = 3). **E.** Glycolysis and glycolytic capacities were calculated based on the data in Figure S6F (n = 3). **F. Measurement of FAO levels in CD8**^+^ **T cells isolated from lung tissues of mice bearing PY8119 (LEV/PLA2G16) tumors, with or without ETO treatment (30 mg kg**^-1^**, i.p.daily).**  (n = 3) **G. The cytokine production from CD8**^+^ **T cells isolated from mice with or without ETO was determined by FACS analysis (n = 7). H. Representative lung** H&E images of mice treated with **or without ETO and quantification of metastasis area** (Scale bars: 100μm) (n = 5). Data are the mean ± SD. *p < 0.05, **p < 0.01, and ***p < 0.001 by two-way ANOVA in (C) and (D) and two-tailed unpaired t test in (others). n.s., not significant.

**
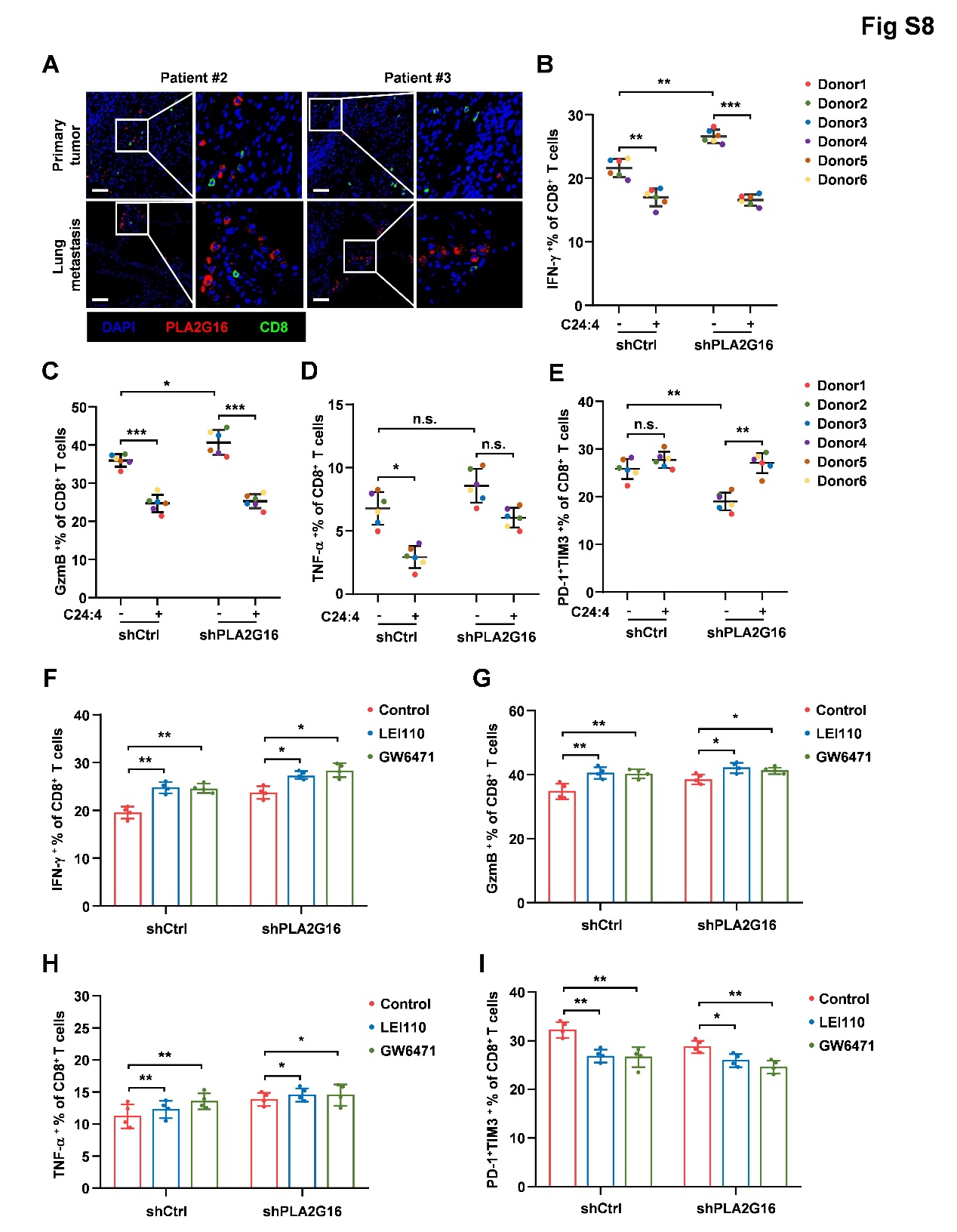
Supplementary Figure 8. C24:4 (n-6) induces dysfunction and impairs cytotoxicity of human T cells**

**A.** Representative IF images of PLA2G16 levels and CD8^+^ T cells in paired primary tumors and lung metastases from breast cancer patients. **B-E.** Flow cytometric quantification of immune effector molecules (B-D) and exhaustion markers (E) of CD8^+^ T cells cultured under the indicated conditions (n=6). **F-I.** Flow cytometric quantification of IFN-γ (F), TNF-α (G), granzyme B (H) and PD-1^+^ TIM-3^+^ (I) in CD8^+^ T cells cultured under the indicated conditions (n = 4). Data are the mean ± SD. *p < 0.05, **p < 0.01, and ***p < 0.001 by two-way ANOVA. n.s., not significant.
