# Supplementary material for: PLA2G16‐Mediated Tetracosatetraenoic Acid Rewires Fatty Acid Oxidation to Impair CD8+ T Cell Immune Function in Promoting Breast Cancer Lung Metastasis
Source: Adv Sci (Weinh). 2025 Nov 16;13(6):e10224. doi: 10.1002/advs.202510224 (PMC12866795; doi:10.1002/advs.202510224)
Supplement: Supplementary file 2 — Supporting Information [file ADVS-13-e10224-s003.pdf]

Supplementary Table 1. Oligomers used in this study

Name	Application	Sequence	
PLA2G16 (Homo)	qRT-PCR	Forward	5’-CCCATTCCAGAGCCTAAGCC-3’
		Reverse	5’-AACCACATATCCATCGCCAAC-3’
Pla2g16 (Mus)	qRT-PCR	Forward	5’-GACGAGGAGTACACCCCACT-3’
		Reverse	5’-CTCACAGTTCTCGCTGGTCA-3’
PPARα (Mus)	qRT-PCR	Forward	5’-AACATCGAGTGTCGAATATGTGG-3’
		Reverse	5’-CCGATAGTTCGCCGAAAGAA-3’
PPARγ (Mus)	qRT-PCR	Forward	5'-GGAAGACCACTCGCATTCCTT-3’
		Reverse	5'-GTAATCAGCAACCATTGGGTCA-3'
PPARδ (Mus)	qRT-PCR	Forward	5’-TCCATCGTCAACAAAGACGGG-3’
		Reverse	5’-ACTTGGGCTCAATGATGTCAC-3’
β-actin (Homo)	qRT-PCR	Forward	5'-GCCGAGGACTTTGATTGC-3’
		Reverse	5'-CCTGTGTGGACTTGGGAGA-3’
β-actin (Mus)	qRT-PCR	Forward	5’-GCTATGCTCTCCCTCACG-3’
		Reverse	5’-ACGCACGATTTCCCTCT-3’
shPLA2G16-1 (Homo)	Knockdown	Target sites	5’-AACUGCGAGCACUUUGUGA-3’
shPLA2G16-2 (Homo)	Knockdown	Target sites	5’-AAGGCCAUCGUGAAGAAGGAA-3’
shPla2g16-1 (Mus)	Knockdown	Target sites	5’-TGGGCCATCTATGTTGGTGAT-3’
shPla2g16-2 (Mus)	Knockdown	Target sites	5’-TACCAGGTCAATAACAAACAT-3’

Supplementary Table 2

PLA2G16 expression in tumor tissues of the breast cancer patient subgroups according to the clinical pathologic parameters in TMA Cohort (140 cases).

Characteristic	All	Low PLA2G16	High PLA2G16	Comparison of PLA2G16 Levels, p value
**Age (years)**				
< 50	72	43	29	
≥ 50	68	46	22	0.3814
**Tumor size (cm)**				
< 3	77	51	26	
≥ 3	63	48	15	0.2628
**Clinical grade**				
I	93	69	24	
II	42	23	19	
III	5	2	3	**0.0354***
**Distal metastasis**				
No	121	82	39	
Yes	19	6	13	**0.0041****
**Vascular invasion**				
No	81	59	22	
Yes	59	36	23	0.1478
**Recurrence**				
No	103	87	16	
Yes	37	23	14	**0.009****

p value was determined by Chip-squared test.
