# Supplementary figures and images for: PLA2G16‐Mediated Tetracosatetraenoic Acid Rewires Fatty Acid Oxidation to Impair CD8+ T Cell Immune Function in Promoting Breast Cancer Lung Metastasis
Source: Adv Sci (Weinh). 2025 Nov 16;13(6):e10224. doi: 10.1002/advs.202510224 (PMC12866795; doi:10.1002/advs.202510224)

**Fig 1F**

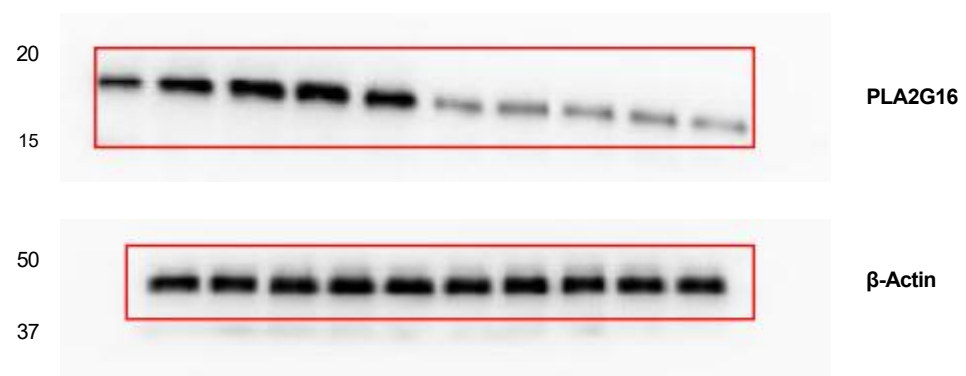

**Fig 6A**

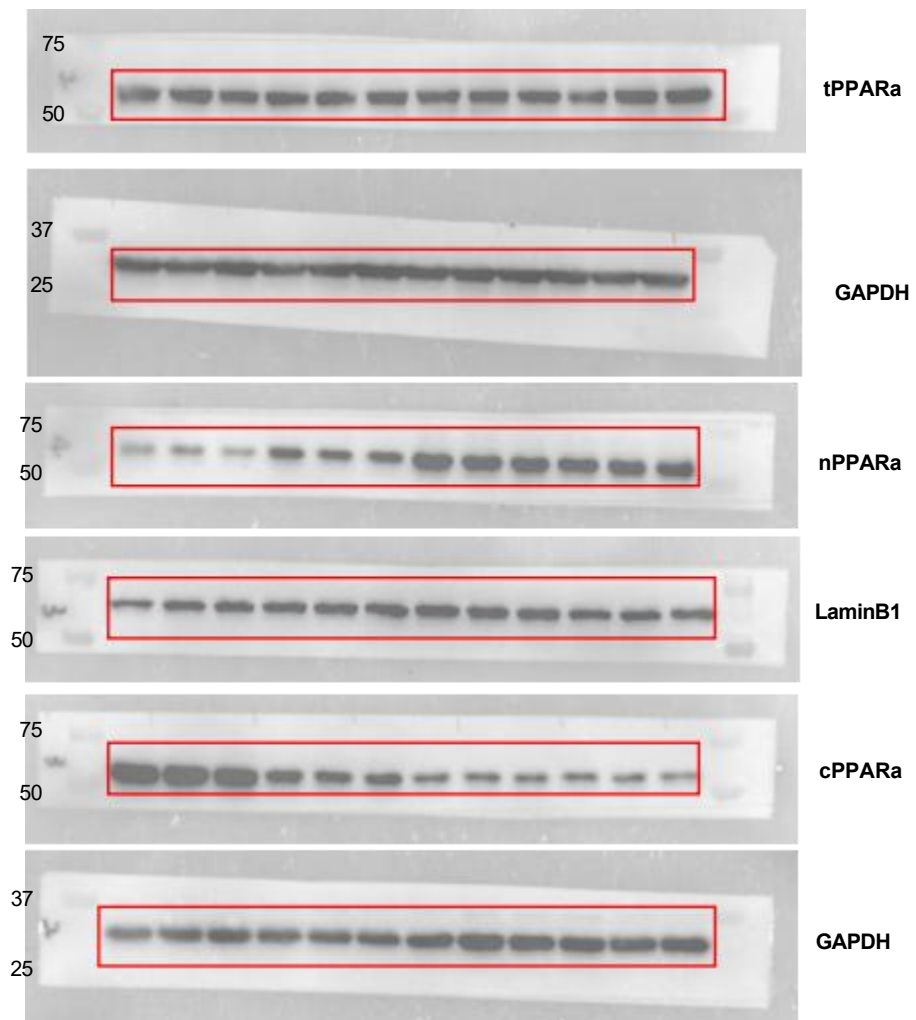

Fig 6B

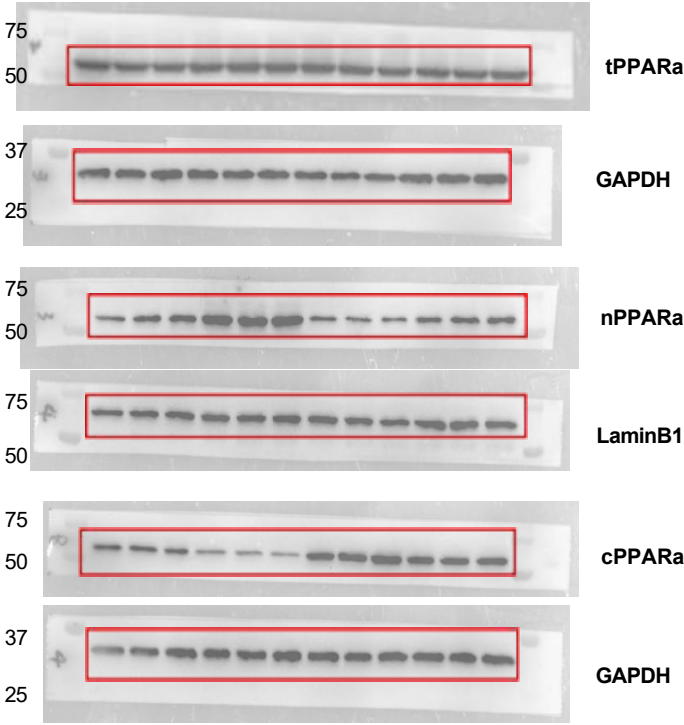

Fig 6C

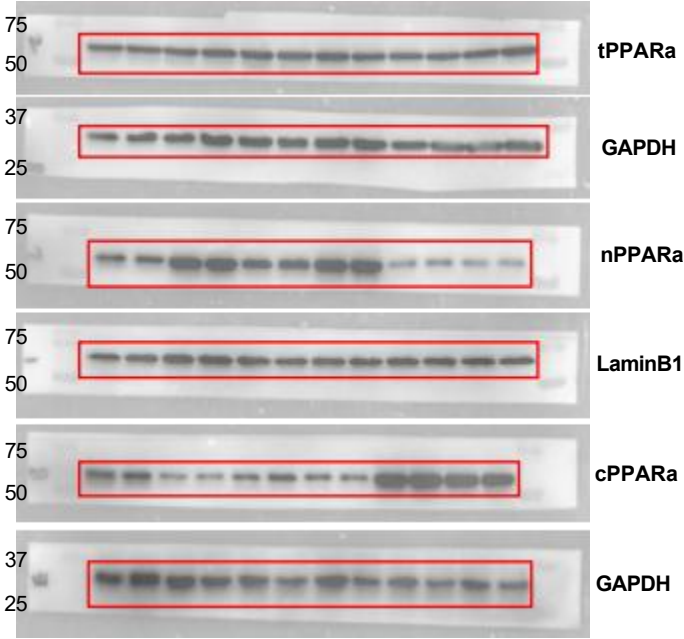

**Fig 6D**

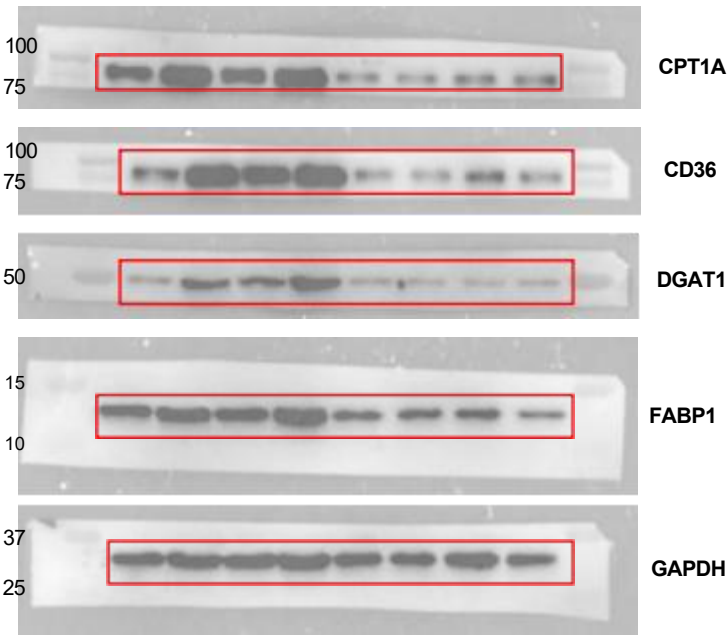

**Fig S1C**

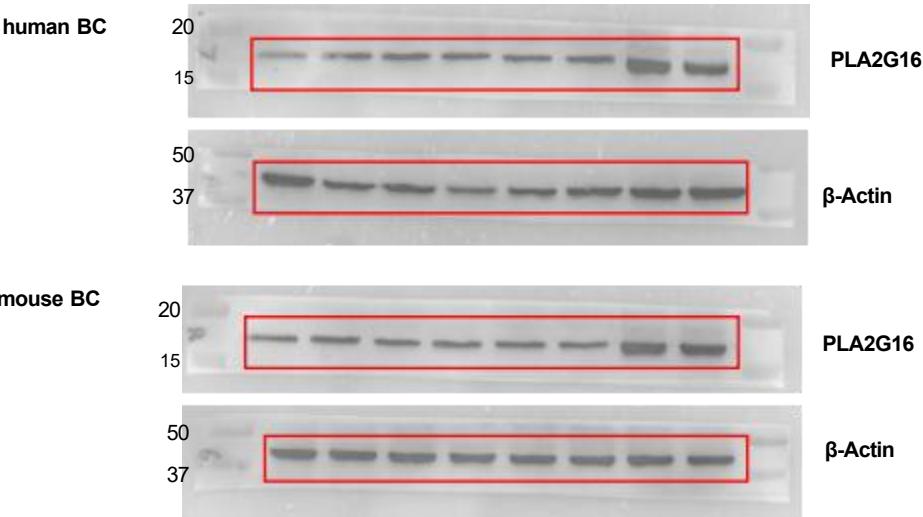

**Fig S1D**

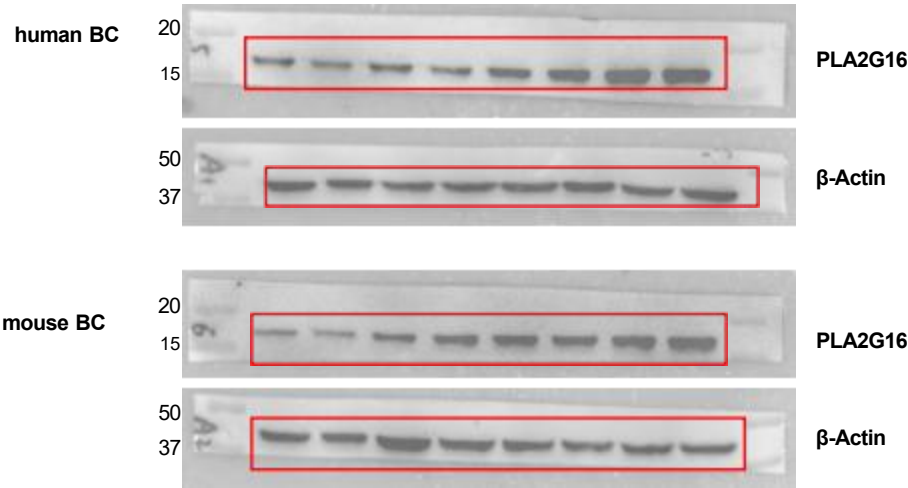

Fig S2C

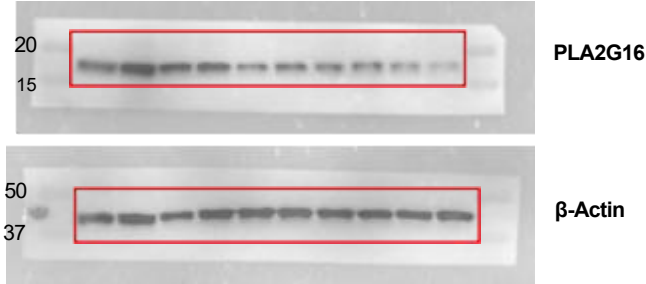

Fig S2D

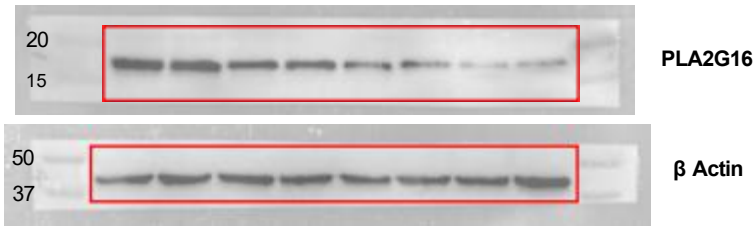

Fig S2E

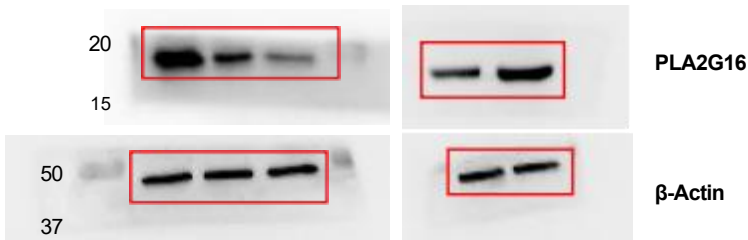

Fig S2F

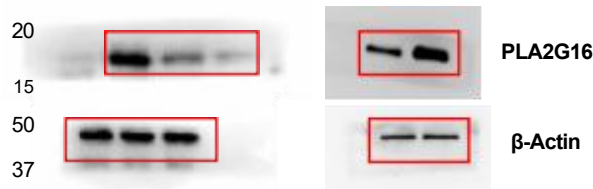

Fig S6C

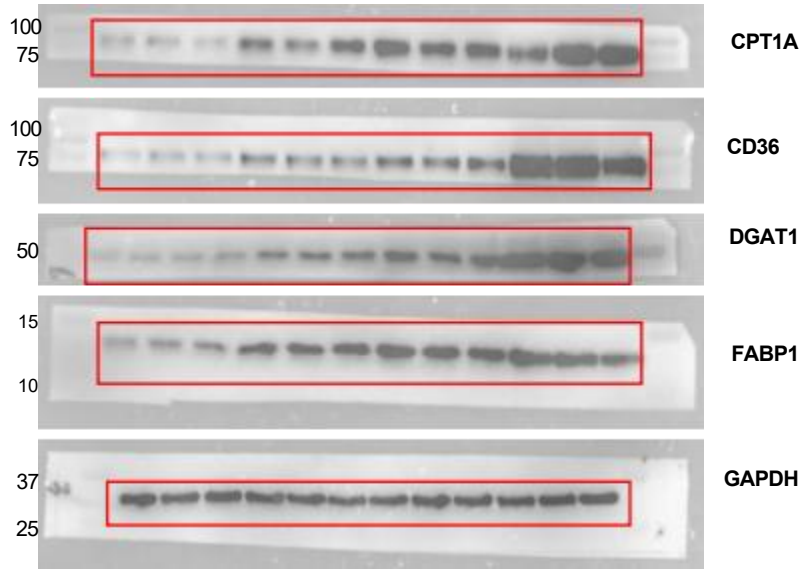

Supplement: Supplementary file 3 — Supporting Information [file ADVS-13-e10224-s001.pdf]
